# Screening of Biomarkers and Quality Control of Shaoyao Gancao Decoction Using UPLC-MS/MS Combined with Network Pharmacology and Molecular Docking Technology

**DOI:** 10.1155/2022/2442681

**Published:** 2022-11-29

**Authors:** Yongben Ma, Lingfang Wu, Liying Niu

**Affiliations:** ^1^Hebei University of Chinese Medicine, Shijiazhuang 050091, China; ^2^Hebei TCM Formula Granule Engineering and Technology Research Center, Shijiazhuang 050091, China; ^3^TCM Quality Evaluation & Reference Compoundization Engineering Research Center, Shijiazhuang 050091, China; ^4^Key Laboratory of Chinese Internal Medicine of Ministry of Education and Beijing, Dongzhimen Hospital, Beijing University of Chinese Medicine, Beijing 100700, China

## Abstract

Shaoyao Gancao Decoction (SGD) is a classic prescription of traditional Chinese medicine (TCM), which is composed of Paeoniae Radix Alba and Glycyrrhizae Radix et Rhizoma, and has the clinical effect of anti-liver injury, but its active ingredients are unclear. In this study, the joint application of phytochemical compositional analysis, network pharmacology, and molecular docking technology was utilized to screen the active components of SGD against liver injury. Firstly, a total of 110 compounds were identified by UPLC-Q-TOF-MS/MS, including 54 flavonoids, 23 triterpenoids, 10 monoterpenoids, 6 coumarins, and 17 other compounds. Secondly, based on the above plant chemical compositions, network pharmacology was used to search for the active components of SGD against liver injury, and 19 components were considered to be the active components, including 1,2,3,4,6-penta-O-galloyl-*β*-D-glucopyranose, ferulic acid, coniferyl ferulate, benzoyl paeoniflorin, hesperidin, liquiritin, liquiritigenin, glycyrrhizic acid, caffeic acid, rutin, chlorogenic acid, gallic acid, methyl gallate, isoliquiritin apioside, albiflorin, neochlorogenic acid, isoliquiritin, narirutin, and naringenin. Thirdly, molecular docking was used to verify the efficacy of the compounds and showed that the compounds bound well to key targets. Furthermore, the 19 components were detected in the rat serum, which also demonstrated that they could be biomarkers. Because it is generally believed that the ingredients that can be absorbed into the blood may be active ingredients. In the end, we determined the contents of 19 key components in 10 different batches of SGD. The method has satisfactory linearity, stability, accuracy, repeatability, and recovery. This study clarified the active components, key targets, and pathways of SGD against liver injury and provided a new idea for the selection of quality control indicators in traditional Chinese medicine.

## 1. Introduction

Shaoyao Gancao Decoction (SGD) is a famous prescription in Zhang Zhongjing's Treatise on Febrile Diseases [1]. It uses Paeonia lactiflora and licorice to nourish yin and blood and to pass through the meridians to treat blood deficiency and body pain, including abdominal pain due to qi and blood deficiency [2–5]. Chemical composition studies show that SGD mainly contains flavonoids, triterpenoid saponins, monoterpenoid glycosides, phenolic acids, tannins, and some other compounds [6–11]. Modern pharmacological studies show that SGD has the functions of relieving spasmolysis and analgesia, protecting the liver, anti-inflammatory, relieving cough and asthma, anti-allergy, and immune regulation, etc. It is clinically applied to spastic pain, liver injury, inflammatory pain, asthma, intestinal ulcers, uterine fibroids, hypertestosterone, nonalcoholic fatty liver, Parkinson's disease, etc. [12–14].

Our previous study found that SGD had an obvious liver-protecting effect [15]. After 14 days of oral administration of SGD in model group rats, the serum alanine aminotransferase (ALT), aspartate aminotransferase (AST), alkaline phosphatase (ALP), and total bilirubin (TBIL) were significantly decreased, and the histopathological structure of the liver was significantly improved. SGD has been widely used in the treatment of viral hepatitis, cholestatic hepatitis, and acute and chronic hepatitis B [16]. The Chinese patent medicine Jianganle Granules prepared from SGD has been used in the clinical treatment of the liver injury caused by various liver diseases and has an obvious liver protection effect [17]. However, the active ingredients and mechanism of SGD against liver injury are still unclear.

This research established a new approach for finding biomarkers for the quality control of SGD. 19 chemical components were selected as the biomarkers of SGD against liver injury based on UPLC-Q-TOF-MS/MS combined with network pharmacology, and molecular docking. And the 19 components were detected in the rat serum, which also proved that they could be biomarkers. In the end, a UPLC-QQQ-MS/MS method for simultaneously determining of the contents of the 19 compounds was constructed. To the best of our knowledge, this is the first report using UPLC-MS/MS, network pharmacology, and molecular docking approaches to find the biomarkers for the quality control of SGD. This study clarified the active components, key targets, and pathways of SGD against liver injury and provided a new idea for the selection of quality control indicators in traditional Chinese medicine. The method developed in our study also provides a scientific foundation for the study of anti-liver injury effective substances in SGD.

## 2. Materials and Methods

### 2.1. Materials and Reagents

Reference substance: 1,2,3,4,6-penta-O-galloyl-*β*-D-glucopyranose (PS020247, purity: 98.0%), coniferyl ferulate (PS011045, purity: 95.0%), benzoylpaeoniflorin (PS000157, purity: 98.0%), methyl gallate (PS020247, purity: 98.0%), isoliquiritin apioside (PS020153, purity: 98.0%), isoliquiritin (PS012517, purity: 98.0%) were purchased from Chengdu Pusi Biotechnology Co., LTD; ferulic acid (110773–201313, purity: 99.6%), hesperidin (110721–201617, purity: 96.1%), liquiritin (111610–201908, purity: 95.0%), caffeic acid (110773–201614, purity: 99.0%), rutin (100080–201811, purity: 91.7%), chlorogenic acid (110753–201817, purity: 96.8%), gallic acid (110831–201204, purity: 89.9%), and narirutin (112007–201602, purity: 90.8%) were purchased from the China National Institute for Food and Drug Control; liquiritigenin (MUST-17022104, purity: 99.07%), glycyrrhizic acid (MUST-17022104, purity: 99.65%), albiflorin (MUST-18041601, purity: 99.14%), neochlorogenic acid (MUST-12113001, purity: 98.00%), and naringenin (MUST-16032406, purity: 99.18%) purchased from Chengdu MUST Biotechnology Co., Ltd. Methanol, formic acid, and acetonitrile (LC-MS grade) were purchased from Merck (Merck & Co., Inc.). Distilled water is supplied by Watsons (A. S. Watson TM Limited).

### 2.2. Sample Preparation

Bai Shao (BS) and Gan Cao (GC) were identified by Sun Baohui, chief pharmacist at the Hebei Drug Inspection Institute. BS is the dry root of Paeonia lactiflora Pall., and GC is the dry root of *Glycyrrhiza* uralensis Fisch. Refer to the original record of “Treatise on Febrile Diseases,” the decocting method of water extract of SGD was determined as follows: take 12 g each of BS and GC, add 600 mL of water, boil to 300 mL, filter, cool the filtrate, concentrate to about 50 mL (1 g: 2 mL) of extract, freeze-dry, and get SGD freeze-dried powder, the average yield of freeze-dried powder is 23%. Precisely weigh 0.1 g of freeze-dried powder, put it in a 25 mL volumetric flask, add 50% methanol to dissolve, sonicate for 10 min, and dilute to the mark with 50% methanol, shake well, and filter with a 0.22 *μ*m filter membrane.

### 2.3. Apparatus and Parameters

#### 2.3.1. Optimization of UPLC-Q-TOF-MS/MS Detection Conditions

A Waters ACQUITY UPLC BEH C_18_ column (100 mm × 2.1 mm, 1.7 *μ*m) was used to separate the aqueous extract of SGD. Take acetonitrile as mobile phase A, and 0.1% aqueous acetic acid as mobile phase B. The gradient elution was as follows: 0 – 3 min, 5% – 15% A; 3 – 5 min, 15% – 18% A; 5 – 10 min, 18% – 24% A; 10 – 14 min, 24% – 26% A; 14 – 25 min, 26% – 45% A; 25 – 29 min, 45% – 100% A; 29 – 30 min, 100% – 5% A; 30 – 35 min, 5% A. The flow rate was 0.3 mL·min^−1^, the column temperature was 35°C, and the injection volume was 1 *μ*L. Mass analysis was performed by Triple TOF TM 6600^+^ (AB SCIEX, Foster City, CA, USA) equipped with an electrospray ionization (ESI) source. MS analysis was performed in both positive and negative ionization modes by full scan mode. The optimized parameters are as follows: ion spray voltage (ISV), 5.5 kV (ESI+) or −4.5 kV (ESI−); ion source temperature (TEM) 550°C; atomized gas 50 psi; auxiliary gas, 50 psi; curtain gas (CUR), 35 psi. The cluster removal potential (DP) is 80 V (ESI+) or −80 V (ESI−). Collision energies (CE) are 40 ± 20 eV (MS mode). TOF-MS scan range: *m*/*z* 100 – 2000. Daughter ion and scanning range: *m*/*z* 50 –1500. In addition, in order to reduce systematic errors and improve the accuracy of mass spectrometry detection, the CDS system was used to calibrate the quality accuracy before each sample injection. When setting up sample batch processing, insert a quality control sample between every three samples to be tested to ensure the accuracy of the test results. The SCIEX OS-Q 2.0 software (AB SCIEX, Foster City, CA, USA) was used for data collection, and the Peak View®2.2 software (AB SCIEX, Foster City, CA, USA) was used for processing and analyzing mass spectrum data.

#### 2.3.2. Optimization of UPLC-QQQ-MS/MS Detection Conditions

UPLC-QQQ-MS/MS analysis was performed on the LC-30A UPLC system (Shimadzu, Kyoto, Japan), including a DGU-30A3 type online vacuum degasser, LC-30AD-type binary pump, SIL-30AC-type automatic sampler, and CTO-30A-type column incubator. The experimental conditions were as follows: Shim-pack GIST C_18_ (100 mm × 2.1 mm, 2 *μ*m) chromatographic column; column temperature, 35°C; flow rate, 0.3 mL·min^−1^; and injection volume, 1 *μ*L. The mobile phase and gradient elution are the same with “2.3.1. Optimization of UPLC-Q-TOF-MS/MS detection conditions.” In terms of mass spectrometry, we chose a QTRAP 4500 (AB SCIEX, Foster City, CA, USA) coupled with an electrospray ionization (ESI) source [18, 19]. The negative was monitored in the scanning mode of multiple reaction monitoring (MRM), the ion source was electrospray ionization (ESI), the ionization voltage (IS) was −4500 V, and the ion source temperature (TEM) was 550°C. The spray gas (GS1, N2) is 345 kPa, the auxiliary gas (GS2, N2) is 345 kPa, the interface is continuously heated, nitrogen gas is introduced throughout the whole process, the curtain gas (CUR, N2) is 207 kPa, and the collision gas (CAD) pressure is medium. The residence time (dwell time) of the ion pair is 50 ms. To optimize transitions, declustering potential (DP), and collision energy (CE) for each component compound, mass spectrometry parameters are shown in Table 1.

### 2.4. Structure Analysis Procedure

By searching the related literature of BS, GC, and SGD, a local database of SGD covering 690 compounds was established, including compound names, molecular formulas, precise relative molecular weights, and mol structures. For the components that could find the reference, we analyzed the structure by comparing the retention time and the secondary mass spectrometry data of the components both in the reference solution and the sample solution. For unknown components, we used OS software to confirm the structure of compounds by comparing the local database of SGD, the TCM MS/MS database, and the online ChemSpider database. Generally, ppm less than 5 is taken as a necessary judgment index. The fragmentation pattern of representative components was analyzed by the MS/MS spectrogram of the compound. The above matching compounds were classified, and the compounds contained in the sample were determined according to the typical MS/MS cracking laws of each type of compound.

### 2.5. Active Ingredient Identification Strategy

Referring to our previous research on the single medicine *Phyllanthus emblica* L. [19], the overall idea was as follows: based on the compounds identified by UPLC-Q-TOF-MS/MS, network pharmacology was used to find the active components of SGD against liver injury. Then molecular docking was used to verify the efficacy of the compounds. In order to further verify the screened biomarkers, we analyzed the chemical components that were absorbed into the rat's blood by SGD. All animal experiments meet the requirements of the Ethics Committee of the Hebei University of Chinese Medicine.

## 3. Results and Discussion

### 3.1. Identification of the Chemical Components in SGD by UPLC-ESI-Q-TOF-MS

**Figure 1:**
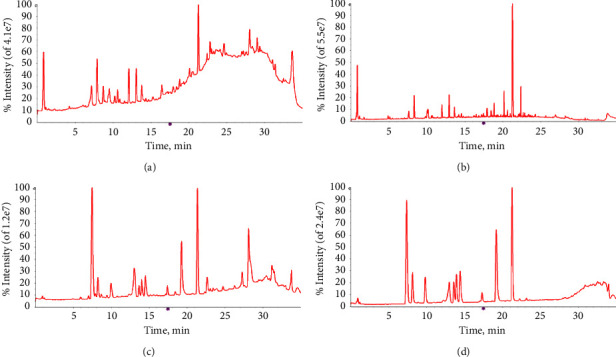
Total ion chromatograms of SGD in positive (a) and negative (b) modes, and total ion chromatograms of reference compound in positive (c) and negative (d) modes.

UPLC-Q-TOF-MS was used to detect the samples in both positive and negative modes. According to reference substance comparison, literature study, and MS/MS information, choose acetonitrile 0.1% acetic acid water as the mobile phase system of good appraisal and concluded that for the 110 chemical elements, the typical total ion chromatograms for positive and negative ion modes are shown in Figure 1. All compounds and related information are shown in Tables 2 and 3.

#### 3.1.1. Identification of Flavonoids

In this study, flavonoids were detected in positive and negative ion modes, respectively, and it was found that the absorption intensity of the tested products was similar in the two modes. 28 compounds were identified in the positive mode, and 26 compounds were identified in the negative mode. It was found that the glycosidic bond was first broken by the cleavage of flavonoid glycosides, and then the chemical bond between sugars was broken. The C-_4_ of the C ring easily lost CO, while the hydroxyl groups of the A ring, B ring, and C ring usually lost H_2_O. CH_2_, CH_4_, or whole branch chains (such as licorice flavone C) were occasionally dropped when there were carbon chains on the ring A. CH_3_ and H_2_O are easily lost when methoxyl and hydroxyl groups replace the B ring. B ring single drop is more common. Cleavage mostly occurs on the C ring, probably due to the carbonyl group on the C ring, the density of the electron cloud is large, easy to occur RDA cleavage. The characteristic ion fragment mass numbers 119 and 137 were found, which can be used as a reference for the identification of flavonoids in the future.

**Figure 2:**
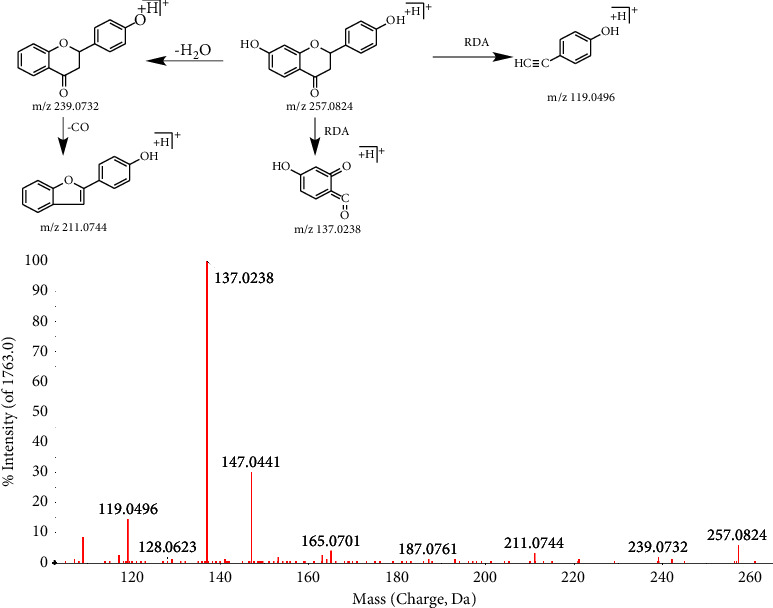
The secondary mass spectrometry of liquiritigenin and its pyrolysis process.

Compound 12^a^ is taken as an example; it shows that the retention time of compound 12 is 9.36 min and the excimer ion peak m/z 257.0824 [M + H]^+^ is formed in the positive mode, and its molecular formula is C_15_H_12_O_4_. With the loss of H_2_O and CO, the fragment ions m/z 239.0732 and 211.0744 were obtained. RDA cleavage produces two ionic fragments, 119.0496 and 137.0238. The secondary mass spectrometry of liquiritigenin and its pyrolysis process are shown in Figure 2.

#### 3.1.2. Identification of Triterpenoids

In this study, a total of 13 triterpenoids were identified in positive mode and 13 in negative mode, mainly pentacyclic triterpenoids. For the pentacyclic triterpenes in positive mode, the A and B rings are easy to break off, followed by the C ring. D and E rings are not easy to break off. However, when the E ring is connected with glucose and other sugars through the glycosidic bond, the glycosidic bond is easy to break off, and the glycosidic bond becomes an aglycone, or two sugars can be directly broken off. In addition, the cleavage of most pentacyclic triterpenoids is confined to the cleavage of the A and B rings. In the negative mode, the glycosidic bond in triterpenoid saponins is basically broken, while the others are similar to the positive mode.

**Figure 3:**
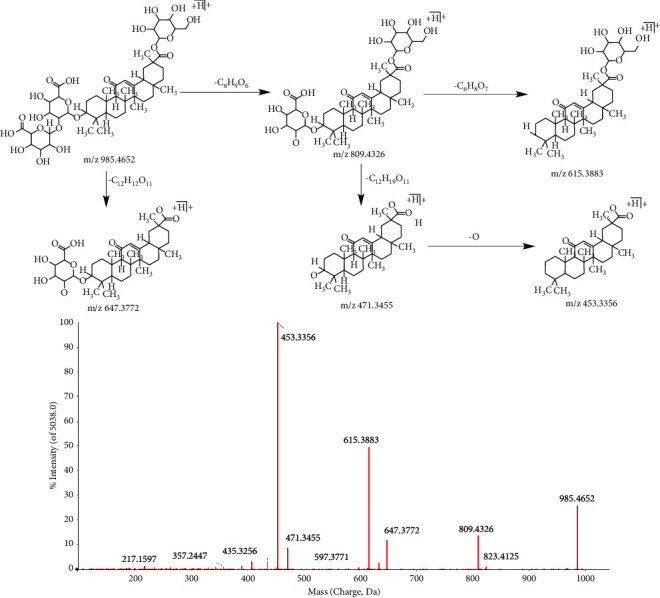
The secondary mass spectrometry of licoricesaponin A3 and its pyrolysis process.

Taking compound 32^b^ as an example, the retention time was 17.58 min, and the excimer ion peak m/z 985.4652 [M + H]^+^ was determined by first-orderfull-scan mass spectrometry in positive mode. The fragment ions m/z 809.4326, 615.3883, 647.3772, 471.3455, and 453.3356 were obtained by the loss of C_6_H_9_O_6_, C_6_H_8_O_7_, C_12_H_12_O_11_, C_12_H_19_O_11_, and C_12_H_20_O_11_. The specific process is shown in Figure 3. The compound was identified as licoricesaponin A3.

#### 3.1.3. Identification of Monoterpene

Six monoterpene glycosides were identified in positive mode, and four were identified in negative mode. In the positive mode, it was found that the ester bond and glycosidic bond of the monoterpene glycosides were broken, the glucosidic and benzyl methyl benzene ketone may continue to dehydroxylation or –O or –CHO cracking for small molecule compounds. Monoterpene glycosides are more difficult to dissociate in the negative mode, and only a few have secondary mass spectrometry, the reason is unclear.

**Figure 4:**
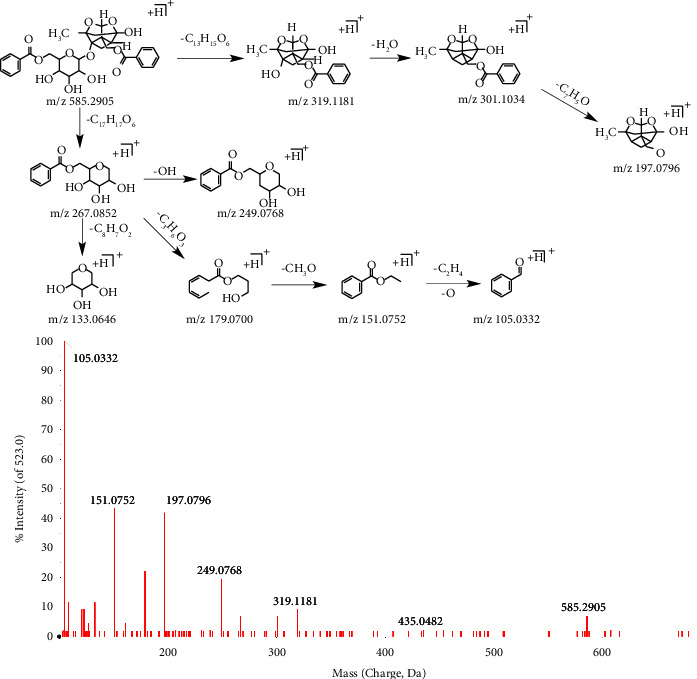
The secondary mass spectrometry of benzoyl paeoniflorin and its pyrolysis process.

Taking compound 31^c^ as an example, the retention time for compound 31^c^ was 17.52 min. In the positive ion mode, first-orderfull-scan mass spectrometry showed the excimer ion peak m/z 585.2905 [M + H]^+^, and second-order scanning mass spectrometry showed that there are fragment ions m/z 319.1181, 301.1034, 197.0796, 267.0852, 249.0768, 133.0646, 179.0700, 151.0752, and 105.0332, as shown in Figure 4. The compound can be identified as benzoyl paeoniflorin.

#### 3.1.4. Identification of Coumarin

In this study, six coumarin compounds were detected in SGD. In the positive mode, according to the secondary fragmentation, the substituents on the pyrone ring of the basic nucleus are very easy to fall off, especially the small groups such as hydroxyl and methoxy, followed by the chain alkane substituents. However, if there is a closed ring substituent on the pyran ring, the stability is increased because the closed ring substituent and the basic core of the compound are coplanar, so it is not easy to crack and fall off. The substituents on the benzene ring of the basic core nucleus are relatively stable. The ring-opening substituents are easier to fall off than the closed-ring substituents. When the benzene ring is connected with an aromatic ring, the aromatic ring may fall off as a whole.

**Figure 5:**
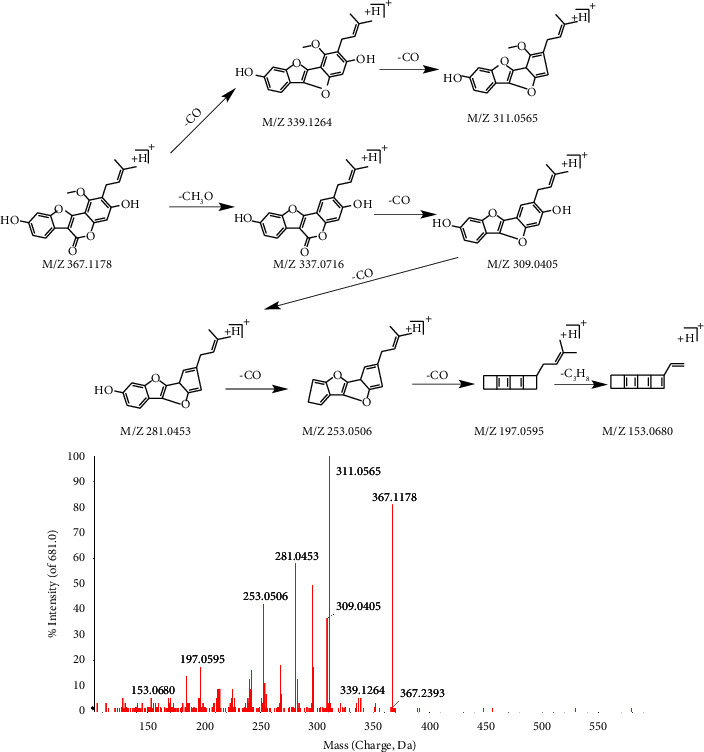
The secondary mass spectrometry of neoglycyrol and its pyrolysis process.

For compound 58^e*∗*^, the retention time was 24.57 min. In positive mode, the first-orderfull-scan mass spectrometry analysis showed that the excemer ion peak m/z 367.1178 [M + H]+, and the second-order scanning mass spectrometry analysis showed that there are fragment ions m/z 339.1264, 337.0716, 309.0405, 281.0453, 253.0506, 197.0595, 153.0680. They are formed by the removal of CO, CH_3_O, C_3_H_8_, and other groups from the parent ion, and the specific process is shown in Figure 5. The compound was further identified as neoglycyrol by reference compound alignment.

#### 3.1.5. Identification of the Other Types

In this study, 17 other compounds were identified, such as aliphatic pentadecanoic acid, and dibutyl phthalate. Aromatic compounds are benzaldehyde, cuminyl acetate, etc. Terpenoids include menthol and so on. If the chemical structure is a chain structure, only alkyl drop will occur. When the ester group is contained, the C–O bond in the ester group is more polar and therefore more likely to break, break off the hydroxyl group, or make its lower molecular weight end fall off.

**Figure 6:**
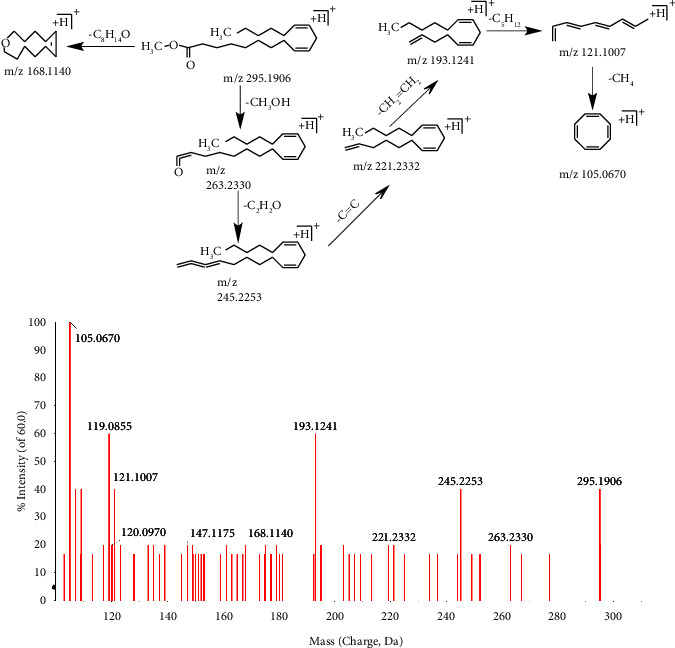
The secondary mass spectrometry of methyl linoleate and its pyrolysis process.

Taking the compound 70^d*∗*^ as an example, the retention time was 31.94 min. In the positive mode, first-orderfull-scan mass spectrometry showed the excimer ion peak m/z 295.1906 [M + H]^+^, and second-order scanning mass spectrometry showed that missing CH_3_OH, C_8_H_14_O, C_2_H_2_O, C = C, CH_2_ = CH_2_, C_5_H_12_, CH_4_, fragment ions m/z 168.1140, 295.1906, 263.2330, 245.2253, 221.2332, 193.1241, 121.1007, and 105.0670 were formed, and the specific process is shown in Figure 6. The compound was further identified as methyl linoleate.

### 3.2. Biomarkers Screening and Validation by Network Pharmacology and Components Absorbed into Blood

**Figure 7:**
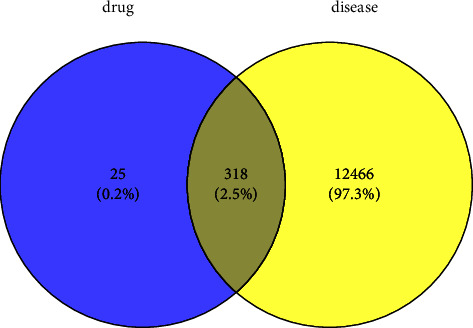
Venn diagram with 318 intersecting targets.

**Figure 8:**
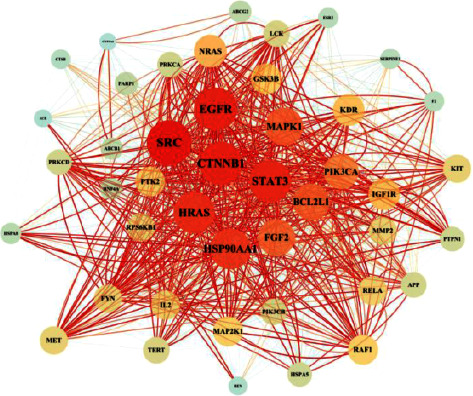
PPI network of 44 genes, the bigger the graph, the bigger the degree. PPI: protein-protein interaction.

**Figure 9:**
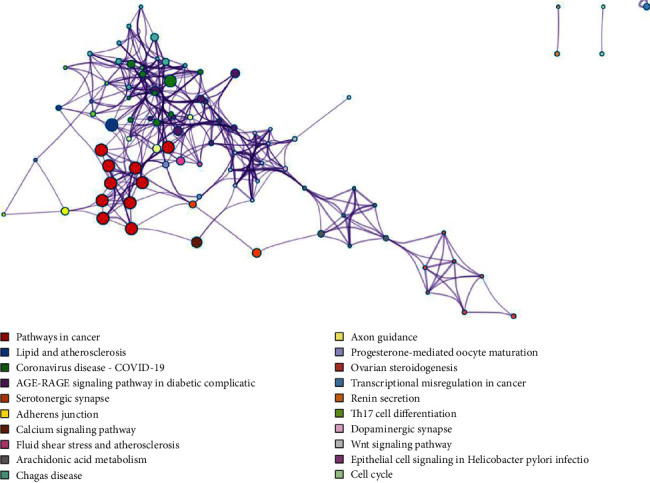
KEGG analysis of potential target genes of the SGD: colored by cluster ID, top 20 clusters of KEGG.

**Figure 10:**
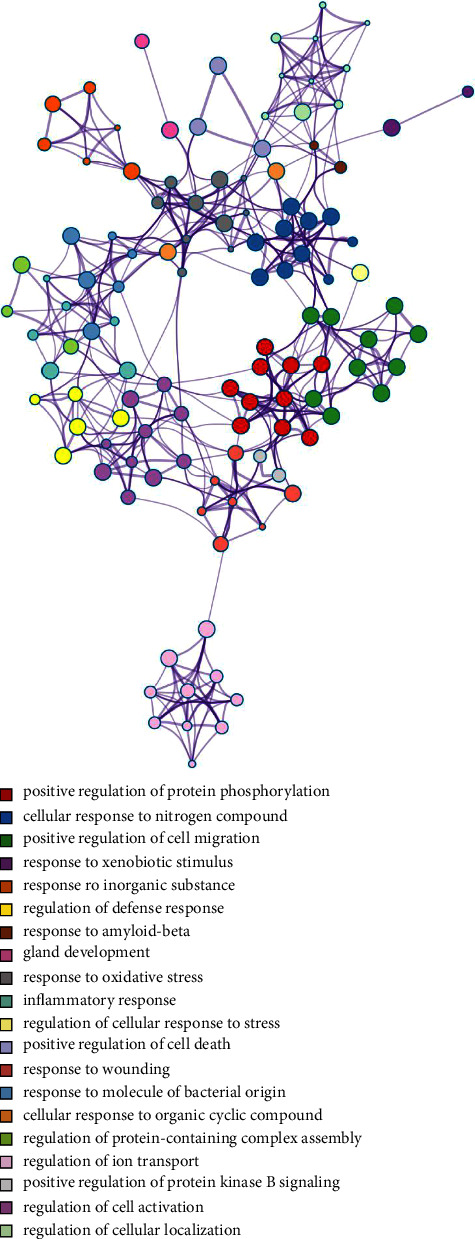
GO analysis of potential target genes of the SGD, colored by cluster ID. Top 20 clusters of GO biological processes.

**Figure 11:**
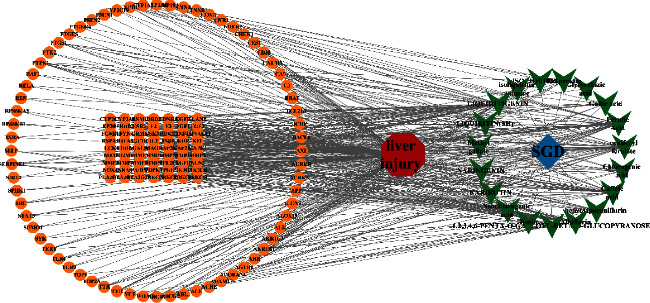
Component target function network diagram.

**Figure 12:**
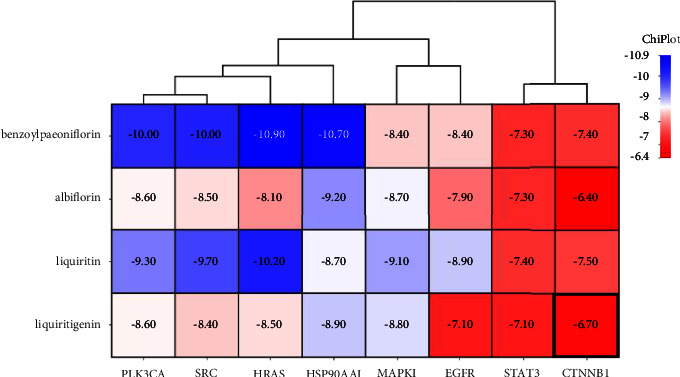
Clustering heatmap of the binding energy of representative compounds and target targets, which can be clustered into three categories.

**Figure 13:**
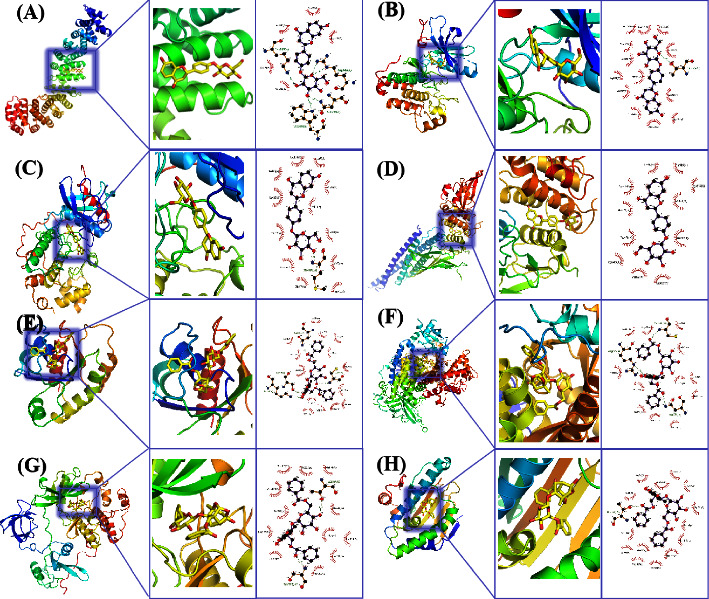
Result of molecular docking between typical chemical composition and targets. (a) Liquiritin with CTNNB1. (b) Liquiritin with EGFR. (c) Liquiritin with MAPK1. (d) Liquiritin with STAT3. (e) Benzoyl paeoniflorin with HRAS. (f) Benzoyl paeoniflorin with PIK3CA. (g) Benzoyl paeoniflorin with SRC. (h) Benzoyl paeoniflorin with HSP90AA1.

A total of 343 potential targets related to the 110 identified compounds were obtained from TCMSP (https://old.tcmsp-e.com/tcmsp.php), SWISS (http://www.swisstargetprediction.ch/), BATMAN (http://bionet.ncpsb.org.cn/batman-tcm/). Through the keyword “liver injury”, a total of 12784 disease targets were obtained from the OMIM (https://omim.org/), Gene Cards (https://www.genecards.org/), and DisGeNET (https://www.disgenet.org/) databases. The Venn diagram is shown in Figure 7. Through protein-protein interaction analysis, 276 targets with a high correlation degree were obtained, and 44 top-ranking genes were obtained after PPI result analysis and screening with a median degree greater than two times, as shown in Figure 8. The relevant parameters of the first 20 targets with a higher degree of integration are shown in Table 4. These 44 retained proteins were further imported into the Clue Go plug-in of the Cytoscape 3.9.1 software for KEGG enrichment analysis, and association analysis with related compounds was conducted. The KEGG pathways of these genes are shown in Figure 9 and Figure 1 in the supplementary materials. The correlation display of the top 20 biological processes (BP) by GO analysis and the GO enrichment analysis obtained the top 10 biological process (BP) items, the top 10 cellular composition (CC) items, and the top 10 molecular functions (MF) entries are shown in Figures 10 and 2 in the supplementary materials. Finally, the “component-target-function” network is visualized in Figure 11, 132 proteins and 19 compounds (including 1,2,3,4,6-penta-O-galloyl-*β*-D-glucopyranose, ferulic acid, coniferyl ferulate, benzoyl paeoniflorin, hesperidin, liquiritin, liquiritigenin, glycyrrhizic acid, caffeic acid, rutin, chlorogenic acid, gallic acid, methyl gallate, isoliquiritin apioside, albiflorin, neochlorogenic acid, isoliquiritin, narirutin, and naringenin) were obtained. Among the 132 proteins and 19 compounds, the EGFR pathway had the highest score and played an important role in the treatment of liver injury, as shown in Table 4. On this basis, we verified it by the molecular docking method. According to the cluster analysis of binding energy, STAT3 and CTNNB1 were clustered into one class; PIK3CA, SRC, HRAS, and HSP90AA1 were clustered into one class, and MAPK1 and EGFR were clustered into one class as shown in Figure 12 and Table 1 in the supplementary materials. The results showed that the docking binding free energies of the 19 compounds were less than −6.4 kcal.mol^−1^. The results of molecular docking between typical chemical compositions are shown in Figure 13 and Table 5. Each type exhibits a similar comprehensive binding capacity with different chemical constituents. All 19 compounds were detected in rat serum by the UPLC-QQQ-MS/MS method, further confirming that these compounds are suitable biomarkers. Detailed information about the analysis of chemical components in rat serum can be found in Table 2 in the supplementary materials.

### 3.3. Validation of the UPLC-QQQ-MS/MS Method

**Figure 14:**
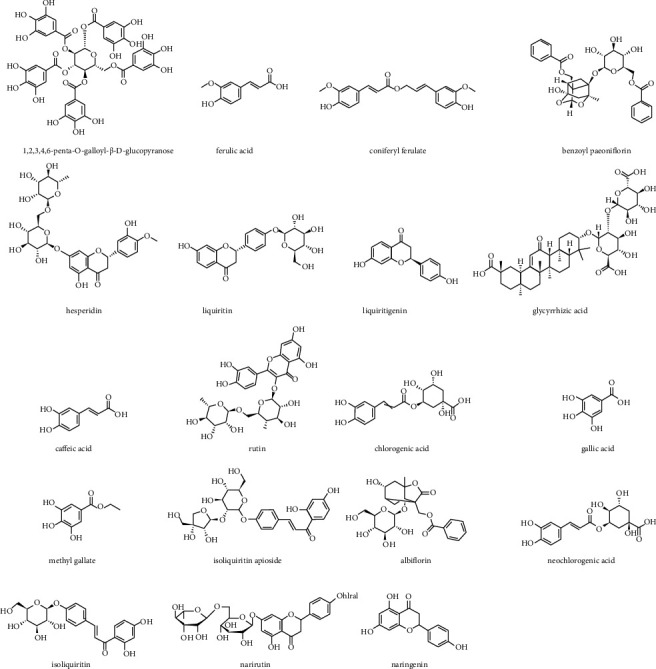
The structures of the 19 compounds.

The stock solution containing the 19 components was prepared. The concentrations of hesperidin, naringenin, liquiritin, glycyrrhizic acid, liquiritigenin, isoliquiritin, isoliquiritin apioside, caffeic acid, ferulic acid, coniferyl ferulate, albiflorin, gallic acid, methyl gallate, benzoyl paeoniflorin, narirutin, chlorogenic acid, neochlorogenic acid, rutin and 1,2,3,4,6-penta-O-galloyl-*β*-D-glucopyranose were 0.98, 0.96, 35.80, 115.50, 13.90, 282.50, 5.00, 19.40, 0.09, 0.15, 0.20, 68.00, 3.30, 0.20, 10.50, 261.70, 0.20, 0.06,0.08, 0.03, 0.025 ng.*μ*L^−1^ The standard curve of the reference substance solution was established, with the concentration (ng.*μ*L^−1^) as the *X*-axis and the peak area as the *Y*-axis. The structures of the 19 compounds are shown in Figure 14. Details of the reference material standard curves are listed in Table 6.

The same sample was injected 6 times continuously to test the precision of the instrument. The RSD values of the peak areas of 19 components were less than 2.05%. It indicated that the precision of the instrument was good.

Inject the same samples at 0, 2, 6, 8, 12, 16, 20, and 24 h, respectively, and determine the peak areas. The RSD of the peak areas of 19 components were all less than 2.11%. The experimental results suggested that the stability of the sample was good within 24 h.

Six samples of the same batch were prepared in parallel, and each sample was injected twice. The RSDs of the contents of 19 components were all less than 2.61%. The experimental results showed that the method had good repeatability.

Take 9 samples of known content samples. Add 50%, 100%, and 150% of the reference solution, respectively. The average recoveries of 19 compounds were 98.11%–103.16%, and the RSD_S_ were all below 2.01%. The experimental results showed that the accuracy of this method was good.

### 3.4. Contents Determination Results of 19 Components

**Figure 15:**
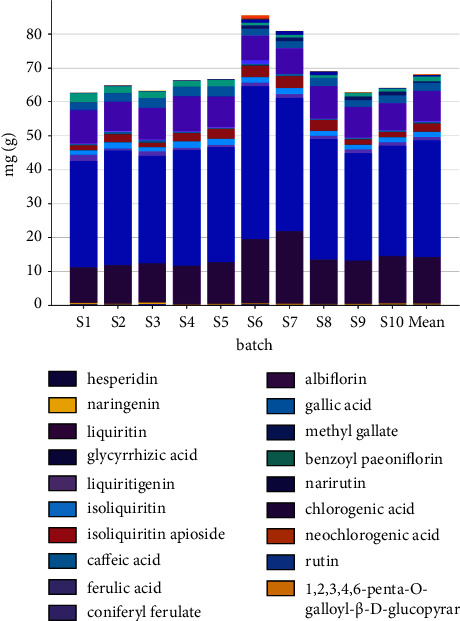
Content histogram of 19 components.

The developed quantitative analysis method was subsequently applied to 10 batches of the SGD. The results demonstrated a successful application of this UPLC-QQQ-MS/MS assay for the quantification of 19 constituents in different samples. The contents, summarized in Table 7 and Figure 15, were calculated with the external reference compound methods. It shows that S6, S7, and S8 have the highest content, while other batches are lower than the median.

In this experiment, UPLC-Q-TOF-MS/MS was employed to analyze SGD. A total of 110 compounds, including 54 flavonoids, 23 triterpenoids, 10 monoterpenoids, 6 coumarins, and 17 other compounds. It can be seen from this result that most of the compounds in SGD are flavonoids, the number of compounds accounted for more than 50% (55/110). From the results of content determination by UPLC-QQQ-MS/MS, the contents of glycyrrhizic acid (content: 3.44%) and liquiritin (content: 1.37%) were significantly higher than those of some flavonoids (all below 1%). These two compounds should be our focus, and furthermore the contents of paeoniflorin and benzoylpaeoniflorin are also relatively high.

## 4. Conclusion

This research established a new method to find biomarkers for the quality control of SGD by the combined application of UPLC-MS/MS, network pharmacology, and molecular docking. Firstly, by comparing the retention times and mass spectrometry dates of the reference database and the self-built database, 110 compounds were identified. Secondly, based on the compounds identified above, network pharmacology was used to find the active components of SGD against liver injury, and 19 chemical components were selected as biomarkers, including 1,2,3,4,6-penta-O-galloyl-*β*-D-glucopyranose, ferulic acid, coniferyl ferulate, benzoyl paeoniflorin, hesperidin, liquiritin, liquiritigenin, glycyrrhizic acid, caffeic acid, rutin, chlorogenic acid, gallic acid, methyl gallate, isoliquiritin apioside, albiflorin, neochlorogenic acid, isoliquiritin, narirutin, and naringenin. Thirdly, molecular docking is used to verify the efficacy of the compounds and shows that the compounds bind well to the key target. Furthermore, the 19 components were detected in the serum, which also proved that they could be biomarkers. Finally, we determined the contents of 19 key components in 10 different batches of SGD. The method has satisfactory linearity, stability, accuracy, repeatability, and recovery. This study clarified the active components, key targets, and pathways of SGD against liver injury and provided a new idea for the selection of quality control indicators of traditional Chinese medicine based on pharmacological activity.

## Figures and Tables

**Table 1:** Mass spectrometry parameters of 19 components.

Analytes	Q1	Q2	DP	CE
Hesperidin	609.1	300.9	−162	−37
Naringenin	270.8	118.6	−153	−32
Liquiritin	417.2	255	−134	−29
Glycyrrhizic acid	821.4	350.8	−206	−55
Liquiritigenin	255	134.7	−118	−25
Isoliquiritin	417.2	254.7	−152	−33
Isoliquiritin apioside	549.1	254.8	−169	−40
Caffeic acid	178.6	134.7	−109	−21
Ferulic acid	193	133.8	−115	−19
Coniferyl ferulate	354.8	192.7	−122	−17
Albiflorin	479.1	120.8	−159	−24
Gallic acid	168.3	124.6	−108	−20
Methyl gallate	182.6	123.7	−113	−29
Benzoyl paeoniflorin	583.9	120.8	−183	−43
Narirutin	579.1	271	−167	−34
Chlorogenic acid	353.2	190.7	−62	−23
Neochlorogenic acid	353.2	190.6	−62	−23
Rutin	609.1	299.8	−184	−43
1,2,3,4,6-penta-O-galloyl-*β*-D-glucopyranose	196.7	123.8	−119	−29

**Table 2:** Positive mode for 70 compounds by UPLC-Q-TOF-MS/MS.

No.	*R*_*t*_ (min)	Formula	Mass (Da)	Ppm	MS/MS fragments	Identification	Source
1^d^	1.48	C_7_H_6_O_5_	170.0215	−1.9	107.0118[M + H-CH_4_O_3_]^+^, 125.0223[M + H-CH_2_O_2_]^+^, 153.0175[M + H-H_2_O]^+^	Gallic acid	BS
2^c^	4.46	C_23_H_28_O_12_	496.1581	−0.8	197.0824[M + H-C_13_H_16_O_8_]^+^; 161.0606[M + H-C_17_H_20_O_7_]^+^; 151.0749[M + H-C_15_H_22_O_9_]^+^; 133.0640[M + H-C_18_H_20_O_8_]^+^; 121.0261[M + H-C_16_H_24_O_10_]^+^	Oxypaeoniflorin	BS
3^a^	4.69	C_15_H_14_O_6_	290.079	−0.1	291.0863[M + H]^+^; 207.0648[M + H-C_4_H_4_O_2_]^+^; 163.0431[M + H-C_6_H_8_O_3_]^+^; 139.0380[M + H-C_8_H_8_O_3_]^+^; 123.0432[M + H-C_8_H_8_O_4_]^+^; 111.0429[M + H-C_9_H_8_O_4_]^+^	Catechin	BS
4^d^	6.62	C_10_H_10_O_4_	194.0579	−1	163.0401[M + H-OCH_4_]^+^, 135.0441[M + H-C_2_H_4_O_2_]^+^, 165.0725[M + H-CH_2_O]^+^	Ferulic acid	BS
5^c^	7.14	C_23_H_28_O_11_	480.1632	0	197.0808[M + H-C_13_H_16_O_7_]^+^; 179.0703[M + H-C_6_H_18_O_6_]^+^; 161.0592[M + H-C_17_H_18_O_6_]^+^; 151.0752[M + H-C_18_H_18_O_6_]^+^; 133.0647[M + H-C_18_H_18_O_7_]^+^; 123.0803[M + H-C_16_H_22_O_9_]^+^; 105.0336[M + H-C_16_H_24_O_10_]^+^	Paeoniflorin	BS
6^c^	7.14	C_23_H_28_O_11_	480.1632	0	197.0808[M + H-C_13_H_16_O_7_]^+^; 179.0703[M + H-C_17_H_18_O_5_]^+^; 161.0592[M + H-C_17_H_10_O_6_]^+^; 151.0752[M + H-C_18_H_18_O_6_]^+^; 133.0647[M + H-C_18_H_20_O_7_]^+^	Albiflorin	BS
7^c^	7.14	C_17_H_18_O_6_	318.1103	0.7	121.0653[M + H-C_10_H_14_O_4_]^+^; 105.0698[M + H-C_10_H_14_O_5_]^+^	Paeoniflorgenin	BS
8^c^	7.14	C_23_H_28_O_11_	480.1632	0	197.0808[M + H-C_13_H_16_O_7_]^+^; 179.0703[M + H-C_17_H_18_O_5_]^+^; 161.0592[M + H-C_17_H_20_O_6_]^+^; 151.0752[M + H-C_18_H_18_O_6_]^+^; 133.0647[M + H-C_18_H_20_O_7_]^+^; 123.0803[M + H-C_16_H_22_O_9_]^+^; 105.0336[M + H-C_16_H_24_O_10_]^+^	Albiflorin R1	BS
9^a^	8.11	C_26_H_28_O_14_	564.1479	−1.8	565.1552[M + H]^+^; 547.1428[M + H-H_2_O]^+^; 529.1343[M + H-H_4_O_2_]^+^; 463.1027[M + H-C_4_H_6_O_3_]^+^; 403.0789[M + H-C_6_H_10_O_5_]^+^; 391.0814[M + H-C_7_H_10_O_5_]^+^; 379.0810[M + H-C_11_H_6_O_3_]^+^; 361.0714[M + H-C_8_H_12_O_6_]^+^; 337.0708[M + H-C_11_H_16_O_5_]^+^; 307.0596[M + H-C_12_H_18_O_6_]^+^	Schaftoside	GC
10^a^	9.08	C_27_H_32_O_14_	580.1792	−0.8	581.1865[M + H]^+^; 257.0813[M + H-C_15_H_16_O_8_]^+^; 239.0679[M + H-C_12_H_22_O_11_]^+^; 147.0438[M + H-C_18_H_26_O_12_]^+^; 137.0244[M + H-C_22_H_20_O_10_]^+^	Glycyrrhizin-7,4′-diglucoside	GC
11^a^	9.08	C_27_H_32_O_14_	580.1792	−0.8	257.0813[M + H-C_12_H_20_O_10_]^+^	Narirutin	BS
12^a^	9.36	C_15_H_12_O_4_	256.0735	0.7	257.0824[M + H]^+^; 147.0441[M + H-C_6_H_6_O_2_]^+^; 137.0238[M + H-C_8_H_8_O]^+^; 119.0496[M + H-C_7_H_6_O_3_]^+^; 109.0284[M + H-C_9_H_8_O_2_]^+^	Liquiritigenin	GC
13^a^	9.52	C_27_H_30_O_14_	578.1635	−1.8	579.1708[M + H]^+^; 543.1475[M + H-H_4_O_2_]^+^; 507.1297[M + H-C_3_H_4_O_2_]^+^; 459.1094[M + H-C_4_H_8_O_4_]^+^; 403.0848[M + H-C_7_H_12_O_5_]^+^; 393.0941[M + H-C_11_H_6_O_3_]^+^; 375.0851[M + H-C_8_H_12_O_6_]^+^; 337.0717[M + H-C_12_H_18_O_5_]^+^; 325.0703[M + H-C_12_H_14_O_6_]^+^	Violanthin	GC
14^b*∗*^	10.59	C_36_H_55_NO_4_	565.4131	12	566.4304[M + H]^+^; 548.4196[M + H-OH_2_]^+^; 209.1654[M + H-C_22_H_31_NO_3_]^+^; 114.0915[M + H-C_31_H_48_O_2_]^+^	Heinsiagenin A	GC
15^d^	10.69	C_41_H_32_O_26_	940.1182	−0.3	455.0820[M + H-C_22_H_14_O_13_]^+^, 345.0208[M + H-C_25_H_24_O_17_]^+^	1,2,3,4,6-penta-O-galloyl-*β*-D-glucopyranose	BS
16^e*∗*^	11.46	C_16_H_12_O_6_	300.0634	−2.2	301.0723[M + H]^+^; 285.0395[M + H-CH_4_]^+;^ 283.0601[M + H-H_2O_]^+;^ 269.0439[M + H-CH_4_O]^+;^ 259.0636[M + H-C_2_H_2_O]^+;^ 257.0436[M + H-CO_2_]^+^; 250.9834[M + H-CH_6_O_2_]^+^; 245.0844[M + H-C_3_H_4_O]^+^; 211.0263[M + H-C_6_H_2_O]^+^; 203.0705[M + H-C_5_H_6_O_2_]^+^; 167.0325[M + H-C_7_H_2_O_3_]^+^; 161.0622[M + H-C_7_H_8_O_3_]^+^; 123.0093[M + H-C_9_H_6_H_4_]^+^; 107.0492[M + H-C_10_H_10_O_4_]^+^	7,2′,4′-trihydroxy-3-(5-meth oxyphenyl) chromen-2-one	GC
17^d*∗*^	11.54	C_12_H_16_O_2_	192.115	2.6	193.0872[M + H]^+^; 149.0244[M + H-C_3_H_6_]^+^	Cuminyl acetate	BS
18^a^	11.72	C_21_H_20_O_10_	432.1056	−1.8	433.2046[M + H]^+^; 271.0596[M + H-C_6_H_10_O_5_]^+^; 133.0875[M + H-C_16_H_12_O_6_]^+^	Vitexin	GC
19^c^	11.74	C_30_H_32_O_15_	632.1741	−1.9	301.1065[M + H-C_13_H_16_O_10_]^+^; 153.0182[M + H-C_23_H_18_O_11_]^+^; 133.0630[M + H-C_25_H_24_O_11_]^+^; 105.0329[M + H-C_23_H_28_O_14_]^+^	Galloyl paeoniflorin	BS
20^a^	11.74	C_28_H_34_O_15_	633.1793	0.5	153.0180[M + Na-C_20_H_25_O_12_]^+^; 105.0329[M + Na-C_24_H_25_H_12_]^+^	Hesperidin	BS
21^a^	11.75	C_15_H_12_O_5_	272.0685	−1	153.0187[M + H-C_7_H_5_O_4_]^+^	Naringenin	GC
22^a^	13.26	C_26_H_30_O_13_	550.1686	−1.4	551.1759[M + H]^+^; 257.0805[M + H-C_11_H_18_O_9_]^+^; 239.0696[M + H-C_11_H_20_O_10_]^+^; 147.0440[M + H-C_17_H_24_O_11_]^+^; 137.0224[M + H-C_19_H_26_O_10_]^+^	Liquiritin apioside	GC
23^a^	13.26	C_26_H_30_O_13_	550.1686	−1.4	551.1759[M + H]^+^; 257.0805[M + H-C_11_H_18_O_9_]^+^; 239.0696[M + H-C_11_H_20_O_10_]^+^; 147.0440[M + H-C_17_H_24_O_11_]^+^; 137.0224[M + H-C_19_H_26_O_10_]^+^	Isoliquiritin apioside	GC
24^a^	13.56	C_21_H_22_O_9_	418.1264	−0.5	419.1337[M + H]^+^; 257.0813[M + H-C_6_H_10_O_5_]^+^; 239.0699[M + H-C_6_H_12_O_6_]^+^; 147.0435[M + H-C_12_H_16_O_7_]^+^; 137.0226[M + H-C_14_H_18_O_6_]^+^	Liquiritin	GC
25^a^	13.56	C_21_H_22_O_9_	418.1264	−0.5	419.1337[M + H]^+^; 257.0813[M + H-C_6_H_10_O_5_]^+^; 239.0699[M + H-C_6_H_12_O_6_]^+^; 147.0435[M + H-C_12_H_16_O_7_]^+^; 137.0226[M + H-C_14_H_18_O_6_]^+^	Isoliquiritin	GC
26^a^	13.76	C_22_H_22_O_9_	430.1264	−0.6	431.1337[M + H]^+^; 269.0809[M + H-C_6_H_10_O_5_]^+^; 253.0484[M + H-C_6_H_10_O_6_]^+^	Ononin	GC
27^a^	13.86	C_16_H_14_O_5_	286.0841	−0.7	287.0925[M + H]^+^; 245.0782[M + H-C_2_H_2_O]^+^; 193.0497[M + H-C_6_H_6_O]^+^; 161.0221[M + H-C_7_H_10_O_2_]^+^; 147.0437[M + H-C_7_H_8_O_3_]^+^; 121.0281[M + H-C_9_H_10_O_3_]^+^; 107.0486[M + H-C_9_H_8_O_4_]^+^	Licochalcone B	GC
28^a^	14.96	C_16_H_12_O_5_	284.0685	−0.1	285.0765[M + H]^+^; 269.0433[M + H-CH_4_]^+^; 253.0504[M + H-CH_4_O]^+^; 241.0484[M + H-C_2_H_4_O]^+^; 213.0545[M + H-C_3_H_4_O_2_]^+^; 185.0581[M + H-C_5_H_8_O_2_]^+^; 137.0226[M + H-C_9_H_8_O_2_]^+^	Calycosin	GC
29^a^	16.83	C_16_H_14_O_4_	270.0892	−0.8	271.0765[M + H]^+^; 229.0868[M + H-C_2_H_2_O]^+^; 177.0548[M + H-C_6_H_6_O]^+^; 147.0429[M + H-C_7_H_8_O_2_]^+^; 123.0433[M + H-C_9_H_8_O_2_]^+^; 121.0285[M + H-C_9_H_10_O_2_]^+^; 107.0497[M + H-C_9_H_8_O_3_]^+^	Retro chalcone	GC
30^e^	17.11	C_21_H_20_O_7_	384.1209	−0.2	385.1291[M + H ]^+^; 367.1194[M + H-OH_2_]^+^; 339.0833[M + H-C_2_H_6_O]^+^; 329.1375[M + H-C_3_H_4_O]^+^; 315.1204[M + H-C_4_H_6_O]^+^; 283.0630[M + H-C_5_H_10_O_2_]^+^; 257.0829[M + H-C_6_H_8_O_3_]^+^; 247.0785[M + H-C_7_H_6_O_3_]^+^; 243.0634[M + H-C_7_H_10_O_3_]^+^; 225.0554[M + H-C_9_H_4_O_3_]^+^	Licopyranocoumarin	BS
31^c^	17.52	C_30_H_32_O_12_	584.1894	−1.2	585.2905[M + H]^+^; 319.1181[M + H-C_13_H_14_O_6_]^+^; 301.1034[M + H-C_13_H_16_O_7_]^+^; 267.0852[M + H-C_17_H_18_O_6_]^+^; 249.0768[M + H-C_17_H_20_O_7_]^+^; 197.0796[M + H-C_20_H_20_O_8_]^+^; 179.0700[M + H-C_20_H_22_O_9_]^+^; 151.0752[M + H-C_21_H_22_O_10_]^+^; 133.0646[M + H-C_25_H_24_O_8_]^+^; 123.0812[M + H-C_23_H_26_O_10_]^+^; 121.0636[M + H-C_23_H_28_O_10_]^+^; 105.0332[M + H-C_23_H_28_O_11_]^+^	Benzoyl paeoniflorin	GC
32^b^	17.58	C_48_H_72_O_21_	985.4566	0.2	985.4652[M + H ]^+^; 809.4326[M + H-C_6_H_8_O_6_]^+^; 647.3772[M + H-C_12_H_18_O_11_]^+^; 615.3883[M + H-C_12_H_18_O_13_]^+^; 471.3455[M + H-C_18_H_26_O_17_]^+^; 453.3356[M + H-C_18_H_28_O_18_]^+^	Licoricesaponin A3	GC
33^a^	19.25	C_16_H_12_O_4_	268.0735	0.7	269.0826[M + H]^+^; 253.0506[M + H-CH_4_]^+^; 237.0555[M + H-CH_4_O]^+^; 225.0555[M + H-C_2_H_4_O]^+^; 213.0925[M + H-C_3_H_4_O]^+^; 137.0238[M + H-C_9_H_8_O]^+^	Formononetin	GC
34^b^	20.06	C_42_H_62_O_17_	838.3987	0.9	484.3436[M + H-C_12_H_16_O_12_]^+^; 469.3328[M + H-C_12_H_18_O_13_]^+^; 451.3219[M + H-C_12_H_20_O_14_]^+^	Licoricesaponin G2	GC
35^b^	20.07	C_30_H_46_O_5_	486.3345	−0.9	487.3432[M + H]^+^; 317.2125[M + H-C_10_H_18_O_2_]^+^	Echinatic acid	GC
36^b^	20.07	C_30_H_46_O_5_	486.3345	−0.9	487.3432[M + H]^+^; 317.2125[M + H-C_10_H_18_O_2_]^+^	Isoechinatic acid	GC
37^b^	20.07	C_30_H_46_O_5_	486.3345	−0.9	487.3432[M + H]^+^; 317.2125[M + H-C_10_H_18_O_2_]^+^	Triphyllic acid	GC
38^b*∗*^	20.07	C_30_H_44_O_4_	468.3239	0	469.3324[M + H]^+^; 451.3214[M + H-H_2_O]^+^; 439.3210[M + H-CH_2_O]^+^	Glabrolide	GC
39^b^	21.23	C_30_H_46_O_4_	470.3396	−0.9	471.3480[M + H]^+^; 317.2118[M + H-C_10_H_18_O]^+^; 235.1689[M + H-C_15_H_24_O_2_]^+^	Glycyrrhetinic acid	GC
40^b^	21.23	C_30_H_46_O_4_	470.3396	−0.9	471.3480[M + H]^+^; 317.2118[M + H-C_10_H_18_O]^+^; 235.1689[M + H-C_15_H_24_O_2_]^+^	Liquiritic acid	GC
41^b*∗*^	21.23	C_30_H_46_O_4_	470.3396	−0.9	471.3480[M + H]^+^; 317.2118[M + H-C_10_H_18_O]^+^; 235.1689[M + H-C_15_H_24_O_2_]^+^	Macedonic acid	GC
42^b^	21.23	C_36_H_54_O_10_	646.3717	−0.2	647.3801[M + H]^+^; 453.3372[M + H-C_6_H_10_O_7_]^+^	Glycyrrhetinic acid 3-O-*β*-D-glucuronide	GC
43^b^	21.23	C_30_H_46_O_4_	471.3469	−0.9	471.3480[M + H]^+^; 317.211[M + H-C_10_H_18_O]^+^; 235.1689[M + H-C_15_H_24_O_2_]^+^	Isomacedonic acid	GC
44^b^	22.16	C_30_H_48_O_3_	456.3604	−1.5	457.3671[M + H]^+^; 303.2310[M + H-C_10_H_18_O]^+^	Oleanolic acid	GC
45^b*∗*^	22.16	C_30_H_48_O_3_	456.3604	−1.5	457.3671[M + H]^+^; 303.2310[M + H-C_10_H_18_O]^+^	Betulinic acid	GC
46^b*∗*^	22.16	C_30_H_48_O_3_	456.3604	−1.5	457.3671[M + H]^+^; 303.2310[M + H-C_10_H_18_O]^+^	11-Deoxyglycyrrhetinic acid	GC
47^b*∗*^	22.16	C_30_H_48_O_3_	456.3604	−1.5	457.3671[M + H]^+^; 303.2310[M + H-C_10_H_18_O]^+^	Glycyrrhetol	GC
48^a^	23.21	C_20_H_20_O_6_	356.12599	0.9	357.1333[M + H]^+^; 301.0710[M + H-C_4_H_8_]^+^; 283.0605[M + H-C_4_H_10_O]^+^; 165.0542[M + H-C_11_H_12_O_3_]^+^; 153.0544[M + H-C_13_H_16_O_2_]^+^; 135.0433[M + H-C_13_H_18_O_3_]^+^; 123.0434[M + H-C_13_H_14_O_4_]^+^	Sigmoidin B	GC
49^d*∗*^	23.21	C_20_H_20_O_6_	356.1259	0.9	301.0710[M + H-C_3_H_4_O]^+^; 191.1066[M + H-C_9_H_10_O_3_]^+^; 167.0698[M + H-C_11_H_10_O_3_]^+^; 153.0544[M + H-C_11_H_12_O_3_]^+^; 137.0592[M - H-C_12_H_11_O_4_]^+^; 135.0433[M + H-C_12_H_14_O_4_]^+^; 123.0434[M + H-C_13_H_14_O_4_]^+^	Piperitol	GC
50^d^	23.21	C_20_H_20_O_6_	356.126	0.9	147.0437[M + H-C_11_H_14_O_4_]^+^, 301.0710[M + H-C_3_H_4_O]^+^, 175.0385[M + H-C_10_H_14_O_3_]^+^, 135.0433[M + H-C_12_H_14_O_4_]^+^	Coniferyl ferulate	GC
51^a*∗*^	23.26	C_17_H_14_O_6_	314.079	0.3	315.0872[M + H]^+^; 299.0563[M + H-CH_4_]^+^; 271.0589[M + H-C_2_H_4_O]^+^; 257.0444[M + H-C_3_H_6_O]^+^; 121.0280[M + H-C_10_H_10_O_4_]^+^	Kumatakenin	GC
52^a^	23.29	C_21_H_20_O_6_	368.1259	0	369.1341[M + H]^+^; 313.0710[M + H-C_4_H_8_]^+^; 285.0763[M + H-C_5_H_8_O]^+^; 271.0600[M + H-C_6_H_10_O]^+^; 243.0656[M + H-C_7_H_10_O_2_]^+^; 227.0700[M + H-C_8_H_12_O_3_]^+^	Licoarylcoumarin	GC
53^e^	23.29	C_21_H_20_O_6_	368.1259	0	369.1341[M + H]^+^; 313.0710[M + H-C_4_H_8_]^+^; 299.1281[M + H-C_5_H_10_]^+^; 285.0763[M + H-C_4_H_4_O_2_]^+^; 283.0606[M + H-C_5_H_10_O]^+^; 271.0600[M + H-C_6_H_10_O]^+^; 257.0800[M + H-C_7_H_12_O]^+^; 243.0656[M + H-C_6_H_6_O_3_]^+^; 227.0700[M + H-C_7_H_10_O_3_]^+^; 209.0584[M + H-C_8_H_4_O_3_]^+^; 205.0495[M + H-C_10_H_12_O_2_]^+^; 181.0632[M + H-C_12_H_14_O_2_]^+^; 135.0436[M + H-C_13_H_14_O_4_]^+^	Glycycoumarin	GC
54^a^	24.14	C_20_H_18_O_6_	354.1103	−0.3	355.1184[M + H]^+^; 337.1076[M + H-H_2_O]^+^; 245.0454[M + H-C_6_H_6_O_2_]^+^; 243.0654[M + H-C_6_H_8_O_2_]^+^; 229.0862[M + H-C_7_H_10_O_2_]^+^; 189.0905[M + H-C_10_H_14_O_2_]^+^; 179.0336[M + H-C_11_H_12_O_2_]^+^; 163.0384[M + H-C_11_H_12_O_3_]^+^; 147.0444[M + H-C_11_H_12_O_4_]^+^	Gancaonin C	GC
55^a^	24.14	C_20_H_18_O_6_	354.1103	−0.3	355.1184[M + H]^+^; 337.1076[M + H-H_2_O]^+^; 299.0556[M + H-C_3_H_4_O]^+^; 217.0500[M + H-C_7_H_6_O_3_]^+^; 201.0910[M + H-C_7_H_6_O4]^+^; 189.0905[M + H-C_8_H_6_O_4_]^+^; 179.0336[M + H-C_11_H_12_O_2_]^+^; 153.0182[M + H-C_13_H_14_O_2_]^+^; 151.0386[M + H-C_11_H_8_O_4_]^+^; 123.0436[M + H-C_14_H_16_O_3_]^+^	Licoisoflavanone	GC
56^a^	24.31	C_21_H_22_O_4_	338.1518	−0.8	339.1577[M + H]^+^; 297.1528[M + H-C_2_H_2_O]^+^; 271.0969[M + H-C_5_H_8_]^+^; 245.1195[M + H-C_6_H_6_O]^+^; 229.0851[M + H-C_7_H_10_O]^+^; 187.0744[M + H-C_8_H_8_O_3_]^+^; 121.0287[M + H-C_14_H_18_O_2_]^+^; 107.0498[M + H-C_14_H_16_O_3_]^+^	Licochalcone A	GC
57^e^	24.4	C_22_H_22_O_6_	382.1416	−0.1	327.0862[M + H-C_4_H_8_]^+^; 311.0545[M + H-C_4_H_8_O]^+^; 299.0918[M + H-C_4_H_4_O_2_]^+^; 297.0383[M + H-C_5_H_10_O]^+^; 295.0595[M + H-C_5_H_12_O]^+^; 283.0605[M + H-C_6_H_12_O]^+^; 163.0396[M + H-C_13_H_16_O_3_]^+^	Glucyrin	GC
58^e*∗*^	24.57	C_21_H_18_O_6_	366.1103	−1	367.1178[M + H]^+^; 339.1264[M + H-CO]^+^; 337.0716[M + H-CH_2_O]^+^; 311.0565[M + H-C_4_H_8_]^+^; 297.0395[M + H-C_5_H_10_]^+^; 283.0615[M + H-C_6_H_12_]^+^; 281.0453[M + H-C_5_H_10_O]^+^; 269.0461[M + H-C_6_H_10_O]^+^; 255.0661[M + H-C_7_H_12_O]^+^	Neoglycyrol	GC
59^a^	24.58	C_20_H_18_O_5_	338.1154	−1	339.1273[M + H]^+^; 297.0762[M + H-C_2_H_2_O]^+^; 283.0604[M + H-C_4_H_8_]^+^; 271.0609[M + H-C_5_H_8_]^+^; 255.0670[M + H-C_5_H_8_O]^+^; 253.0456[M + H-C_5_H_10_O]^+^; 227.0678[M + H-C_6_H_8_O_2_]^+^	Licoflavone C	GC
60^a^	24.6	C_21_H_22_O_5_	354.1467	−0.7	355.1467[M + H]^+^; 299.0934[M + H-C_3_H_4_O]^+^; 215.1055[M + H-C_7_H_8_O_3_]^+^; 191.1081[M + H-C_9_H_8_O_3_]^+^; 173.0974[M + H-C_9_H_10_O_4_]^+^; 153.0550[M + H-C_13_H_14_O_2_]^+^; 135.0434[M + H-C_12_H_12_O_4_]^+^; 123.0460[M + H-C_14_H_16_O_3_]^+^; 107.0489[M + H-C_14_H_16_O_4_]^+^	3′-methoxyglabridin	GC
61^a^	25.17	C_20_H_20_O_4_	324.1361	−0.6	325.1362[M + H]^+^; 189.0905[M + H-C_8_H_8_O_2_]^+^; 149.0597[M + H-C_11_H_12_O_2_]^+^; 147.0812[M + H-C_11_H_14_O_2_]^+^; 123.0434[M + H-C_13_H_14_O_2_]^+^; 121.0648[M + H-C_12_H_12_O_3_]^+^	Bavachin	GC
62^a^	25.38	C_21_H_20_O_5_	352.131	−0.9	353.1399[M + H]^+^; 325.1398[M + H-CO]^+^; 323.0944[M + H-CH_2_O]^+^; 193.0485[M + H-C_11_H_12_O]^+^; 189.0923[M + H-C_9_H_8_O_3_]^+^; 165.0548[M + H-C_12_H_12_O_2_]^+^; 147.0799[M + H-C_12_H_14_O_3_]^+^; 135.0463[M + H-C_13_H_14_O_3_]^+^; 123.0438[M + H-C_13_H_10_O_4_]^+^; 119.0494[M + H-C_13_H_14_O_4_]^+^	Gancaonin G	GC
63^a^	25.55	C_20_H_16_O_6_	352.0947	−1.3	353.1023[M + H]^+^; 335.0916[M + H-H_2_O]^+^; 325.1064[M + H-CO]^+^; 311.0553[M + H-C_3_H_6_]^+^; 295.0596[M + H-C_3_H_6_O]^+^; 283.0596[M + H-C_5_H_10_]^+^; 217.0489[M + H-C_7_H_4_O_3_]^+^; 153.0176[M + H-C_13_H_12_O_2_]^+^	Licoisoflavone B	GC
64^a^	26.03	C_20_H_16_O_5_	336.0997	−0.5	337.1053[M + H]^+^; 321.0773[M + H-CH_4_]^+^; 319.0968[M + H-H_2_O]^+^; 309.1153[M + H-CO]^+^; 295.0589[M + H-C_3_H_6_]^+^; 271.0615[M + H-C_5_H_6_]^+^; 267.0651[M + H-C_4_H_6_O]^+^; 253.0488[M + H-C_5_H_8_O]^+^	Glabrone	GC
65^a*∗*^	26.48	C_22_H_26_O_5_	370.178	1	371.1853[M + H]^+^; 315.1234[M + H-C_4_H_8_]^+^; 303.1230[M + H-C_5_H_8_]^+^; 235.1331[M + H-C_8_H_8_O_2_]^+^; 193.0861[M + H-C_11_H_14_O_2_]^+^; 167.0703[M + H-C_13_H_16_O_2_]^+^; 149.0596[M + H-C_13_H_18_O_3_]^+^; 137.0602[M + H-C_14_H_18_O_3_]^+^; 123.0442[M + H-C_15_H_20_O_3_]^+^	Glyasperin D	BS
66^d^	28.01	C_16_H_22_O_4_	278.1518	−1	149.0237[M + H-C_8_H_18_O]^+^; 121.0290[M + H-C_9_H_18_O_2_]^+^	Disobutyl phthalate	BS
67^d^	28.01	C_16_H_22_O_4_	278.1518	−1	149.0237[M + H-C_8_H_18_O]^+^; 121.0290[M + H-C_9_H_18_O_2_]^+^	Dibutyl phthalate	BS
68^d*∗*^	28.53	C_18_H_32_O_2_	280.2402	−1.4	178.9986[M + H-C_5_H_10_O_2_]^+^; 151.0303[M + H-C_7_H_14_O_2_]^+^; 149.1343[M + H-C_7_H_16_O_2_]^+^; 141.0669[M + H-C_8_H_14_O_2_]^+^; 121.1009[M + H-C_9_H_13_]^+^; 109.1069[M + H-C_8_H_13_]^+^	Linoleic acid	BS
69^d*∗*^	31.53	C_15_H_30_O_2_	242.2246	0	243.115[M + H]^+^; 173.0611[M + H-C_5_H_10_]^+^; 143.0849[M + H-C_7_H_16_]^+^; 141.0683[M + H-C_5_H_10_O_2_]^+^; 129.0689[M + H-C_8_H_18_]^+^; 115.0550[M + H-C_9_H_20_]^+^	Pentadecanoic acid	BS
70^d*∗*^	31.94	C_19_H_34_O_2_	294.2559	−1	295.1906[M + H]^+^; 263.2330[M + H-CH_4_O]^+^; 221.2332[M + H-C_3_H_6_O_2_]^+^; 219.2069[M + H-C_3_H_8_O_2_]^+^; 193.1241[M + H-C_5_H_10_O_2_]^+^; 123.1224[M + H-C_9_H_20_O_2_]^+^	Methyl linoleate	

a: flavonoids, b: triterpenoids, c: monoterpenoid glycosides, d: other components, e: coumarins, *∗* indicates the first discovered component.

**Table 3:** Negative mode for 53 compounds by UPLC-Q-TOF-MS/MS.

No	*R*_*t*_ (min)	Formula	Mass (Da)	Ppm	MS/MS fragments	Identification	Source
71^d^	3.51	C_9_H_8_O_4_	180.0423	−3.9	134.9873[M-H-CH_2_O_2_]^–^	Caffeic acid	GC
72^d^	3.88	C_9_H_10_O_5_	198.0528	−2.6	152.5018[M-H-C_2_H_6_O]^–^	Ethyl gallate	BS
73^d^	4.09	C_8_H_8_O_5_	184.0372	−3.4	123.0118[M-H-C_2_H_6_O_2_]^–^	Methyl gallate	BS
74^c^	5.45	C_23_H_28_O_12_	496.158	−1	495.1509[M-H]^–^; 465.1404[M-H-CH_2_O]^–^; 333.1006[M-H-C_6_H_10_O_5_]^–^; 195.0680[M-H-C_13_H_16_O_8_]^–^; 177.0547[M-H-C_17_H_18_O_6_]^–^; 165.0554[M-H-C_14_H_18_O_9_]^–^; 137.0242[M-H-C_16_H_22_O_9_]^–^	Oxy paeoniflorin	BS
75^a^	7.51	C_21_H_20_O_11_	448.1005	−0.2	447.0961[M-H]^–^; 285.0405[M-H-C_6_H_10_O_5_]^–^; 265.8768[M-H-C_6_H_14_O_6_]^–^; 130.9660[M-H-C_16_H_12_O_7_]^–^; 109.0305[M-H-C_15_H_14_O_9_]^–^	Kaempferol-3-O-*β*-D-glucoside	BS
76^a^	7.51	C_21_H_20_O_11_	448.1005	−0.2	447.0961[M-H]^–^; 285.0405[M-H-C_6_H_10_O_5_]^–^; 265.8768[M-H-C_6_H_14_O_6_]^–^; 130.9660[M-H-C_16_H_12_O_7_]^–^	Kaempferol-7-O-*β*-D-glucoside	BS
77^c^	8.32	C_23_H_28_O_11_	480.1631	−0.6	327.1093[M-H-C_8_H_8_O_3_]^–^; 167.0562[M-H-C_14_H_18_O_8_]^–^; 121.0299[M-H-C_16_H_22_O_9_]^–^	Albiflorin	BS
78^d^	8.32	C_16_H_18_O_9_	309.0969	0.2	309.0969[M-COOH]^–^	Chlorogenic acid	GC
79^d^	8.32	C_16_H_18_O_9_	309.0969	0.2	309.0969[M-COOH]^–^	Neochlorogenic acid	BS
80^a^	8.8	C_27_H_30_O_14_	578.1635	0.4	577.1589[M-H]^–^; 487.1256[M-H-C_3_H_6_O_3_]^–^; 473.1098[M-H-C_4_H_8_O_3_]^–^; 457.1160[M-H-C_4_H_8_O_4_]^–^; 413.0884[M-H-C_6_H_12_O_5_]^–^; 395.0789[M-H-C_6_H_14_O_6_]^–^	Isoviolanthin	BS
81^c^	11.51	C_16_H_24_O_8_	344.357	−2.4	208.8396[M-H-C_5_H_10_O_4_]^–^; 165.0559[M-H-C_6_H_10_O_6_]^–^; 101.0234[M-H-C_12_H_18_O_5_]^–^	Mudanpioside F	GC
82^a^	12.21	C_27_H_30_O_16_	609.1440	−3.4	609.1440[M-H]^–^	Rutin	BS
83^a^	14.11	C_21_H_20_O_12_	464.0955	−1.3	463.0877[M-H]^–^; 417.0979[M-H-CH_2_O_2_]^–^; 265.1488[M-H-C_6_H_14_O_7_]^–^; 174.9527[M-H-C_12_H_16_O_8_]^–^; 130.9658[M-H-C_16_H_12_O_8_]^–^	Quercetin-3-O-*β*-D-glucoside	GC
84^a^	14.37	C_16_H_12_O_5_	284.0685	2.7	283.0629[M-H]^–^; 267.0298[M-H-CH_4_]^–^; 239.0352[M-H-C_2_H_4_O]^–^; 211.0397[M-H-C_3_H_4_O_2_]^–^; 183.0457[M-H-C_5_H_8_O_2_]^–^; 135.0083[M-H-C_9_H_8_O_2_]^–^	Calycosin	GC
85^c^	14.43	C_30_H_32_O_14_	616.1792	−1.7	615.1719[M-H]^–^; 597.1627[M-H-H_2_O]^–^; 493.1346[M-H-C_7_H_6_O_2_]^–^; 475.1250[M-H-C_7_H_8_O_3_]^–^; 376.8781[M-H-C_11_H_10_O_6_]^–^; 313.0538[M-H-C_6_H_14_O_6_]^–^; 174.9550[M-H-C_6_H_7_O_6_]^–^; 151.0032[M-H-C_8_H_7_O_3_]^–^	Mudanpioside H	GC
86^b^	14.82	C_36_H_38_O_16_	726.2159	1	725.2122[M-H]^–^; 549,1637[M-H-C_10_H_8_O_3_]^–^; 531.1518[M-H-C_10_H_10_O_4_]^–^; 417.1194[M-H-C_15_H_16_O_7_]^–^; 399.1085[M-H-C_15_H_18_O_8_]^–^; 255.0668[M-H-C_21_H_26_O_12_]^–^; 193.0504[M-H-C_16_H_28_O_12_]^–^; 175.0402[M-H-C_26_H_30_O_13_]^–^; 135.0091[M-H-C_29_H_34_O_13_]^–^	Licorice glycoside C2	GC
87^a*∗*^	15.22	C_16_H_10_O_6_	298.0477	1.7	297.0421[M-H]^–^; 197.0259[M-H-C_5_H_8_O_2_]^–^	Isotrifoliol	GC
88^e*∗*^	15.22	C_16_H_12_O_6_	300.0634	−0.6	299.0565[M-H]^–^; 255.0305[M-H-CO_2_]^–^; 199.0415[M-H-C_5_H_8_O_2_]^–^	7,2′,4′-trihydroxy-3-(5-meth oxy phenyl) chromen-2-one	GC
89^a^	15.83	C_27_H_30_O_13_	562.1686	0.9	561.1614[M-H]^–^; 267.0668[M-H-C_11_H_18_O_9_]^–^	Glycyroside	GC
90^a^	16.48	C_20_H_18_O_7_	370.1053	0.5	369.1000[M-H]^–^; 243.0681[M-H-C_6_H_6_O_3_]^–^; 219.0674[M-H-C_7_H_2_O_4_]^–^; 191.0724[M-H-C_11_H_14_O_2_]^–^; 175.0753[M-H-C_9_H_6_O_5_]^–^; 151.0048[M-H-C_13_H_14_O_3_]^–^; 125.0266[M-H-C_14_H_12_O_4_]^–^; 109.0289[M-H-C_13_H_8_O_6_]^–^; 107.0154[M-H-C_14_H_14_O_4_]^–^	Uralenol	GC
91^a^	17.29	C_16_H_12_O_4_	268.0735	0.9	267.0674[M-H]^–^; 251.0359[M-H-CH_4_]^–^; 223.0404[M-H-C_2_H_4_O]^–^; 135.0083[M-H-C_9_H_8_O]^–^	Formononetin	GC
92^b^	17.92	C_48_H_72_O_21_	985.073	1.5	983.4542[M-H]^–^; 821.3998[M-H-C_6_H_10_O_5_]^–^	Licoricesaponin A3	GC
93^a^	18.71	C_15_H_12_O_4_	256.0735	2.2	255.0663[M-H]^–^; 119.0508[M-H-C_7_H_4_O_3_]^–^; 135.0093[M-H-C_8_H_8_O]^–^	Pinocembrin	GC
94^a^	19.93	C_16_H_14_O_4_	270.0892	1.2	269.0817[M-H]^–^; 253.0532[M-H-CH_4_]^–^; 237.0538[M-H-CH_4_O]^–^; 225.0596[M-H-C_2_H_4_O]^–^; 175.0408[M-H-C_6_H_6_O]^–^; 161.0248[M-H-C_7_H_8_O]^–^; 149.0240[M-H-C_7_H_4_O_2_]^–^; 133.0289[M-H-C_8_H_8_O_2_]^–^; 117.0352[M-H-C_8_H_8_O_3_]^–^; 107.0128[M-H-C_10_H_10_O_2_]^–^; 105.0342[M-H-C_9_H_8_O_3_]^–^	Retrochalcone	GC
95^b^	20.17	C_42_H_60_O_16_	820.3881	0.5	819.3884[M-H]^–^; 351.0593[M-H-C_36_H_44_O_4_]^–^	Licoricesaponin E2	GC
96^b^	20.3	C_50_H_76_O_21_	1012.2879	0.3	1011.4853[M-H]^–^; 497.1146[M-H-C_38_H_50_O_5_]^–^; 146.9654[M-H-C_50_H_64_O_17_]^–^	Licoricesaponin D3	GC
97^b^	21.28	C_42_H_62_O_16_	822.4038	2.3	821.4106[M-H]^–^; 351.0577[M-H-C_30_H_46_O_4_]^–^; 193.0363[M-H-C_6_H_9_O_7_]^–^	Glycyrrhizic acid	GC
98^b^	21.28	C_42_H_62_O_16_	822.5038	2.3	821.4106[M-H]^–^; 351.0577[M-H-C_30_H_46_O_4_]^–^; 193.0363[M-H-C_6_H_9_O_7_]^–^	Licoricesaponin H2	BS
99^b^	21.28	C_42_H_62_O_16_	822.4038	2.3	821.4106[M-H]^–^; 351.0577[M-H-C_30_H_46_O_4_]^–^; 193.0363[M-H-C_6_H_9_O_7_]^–^	Licoricesaponin K2	GC
100^a^	21.6	C_21_H_20_O_6_	368.1259	−0.2	367.1196[M-H]^–^; 337.0718[M-H-CH_2_O]^–^; 309.0416[M-H-C_3_H_6_O]^–^; 297.0409[M-H-C_5_H_10_]^–^; 281.0456[M-H-C_5_H_10_O]^–^; 265.0507[M-H-C_6_H_14_O]^–^; 203.0712[M-H-C_9_H_8_O_3_]^–^	Licoarylcoumarin	GC
101^d*∗*^	22.79	C_12_H_16_O_2_	192.115	−3.5	190.8612[M-H]^–^; 162.8911[M-H-C_2_H_4_]^–^; 102.9487[M-H-C_4_H_8_O_2_]^–^	Cuminyl acetate	GC
102^a^	23.02	C_20_H_20_O_4_	324.1362	2.9	323.1280[M-H]^–^; 213.0929[M-H-C_6_H_6_O_2_]^–^; 187.0765[M-H-C_8_H_8_O_2_]^–^; 185.0609[M-H-C_8_H_10_O_2_]^–^; 175.0754[M-H-C_9_H_8_O_2_]^–^; 147.0446[M-H-C_11_H_12_O_2_]^–^; 135.0455[M-H-C_13_H_16_O]^–^; 107.0502[M-H-C_14_H_16_O_2_]^–^	Isobavachalcone	GC
103^a^	23.02	C_20_H_20_O_4_	324.1362	2.9	323.1280[M-H]^–^; 213.0929[M-H-C_6_H_6_O_2_]^–^; 201.0922[M-H-C_7_H_6_O_2_]^–^; 187.0765[M-H-C_8_H_8_O_2_]^–^; 175.0754[M-H-C_9_H_8_O_2_]^–^; 147.0446[M-H-C_11_H_12_O_2_]^–^; 135.0455[M-H-C_13_H_16_O]^–^; 107.0502[M-H-C_14_H_16_O_2_]^–^	Glabridin	GC
104^a^	23.38	C_20_H_18_O_6_	354.1103	1.9	353.1046[M-H]^–^; 285.1144[M-H-C_5_H_8_]^–^; 267.1041[M-H-C_5_H_10_O]^–^; 243.1037[M-H-C_6_H_6_O_2_]^–^; 201.0925[M-H-C_9_H_12_O_2_]^–^	Licoflavonol	GC
105^a^	23.38	C_20_H_18_O_6_	354.1103	1.9	353.1046[M-H]^–^; 285.1144[M-H-C_5_H_8_]^–^; 267.1041[M-H-C_5_H_10_O]^–^; 243.1037[M-H-C_6_H_6_O_2_]^–^; 227.0720[M-H-C_6_H_6_O_3_]^–^; 201.0925[M-H-C_9_H_12_O_2_]^–^; 125.0250[M-H-C_14_H_12_O_3_]^–^	Glycyrrhisoflavone	GC
106^d*∗*^	23.38	C_20_H_20_O_6_	356.1259	0	355.1179[M-H]^–^; 203.1076[M-H-C_8_H_8_O_3_]^–^; 165.0551[M-H-C_11_H_10_O_3_]^–^	Piperitol	GC
107^e^	23.41	C_21_H_20_O_6_	368.1259	−0.2	367.1196[M-H]^–^; 337.0718[M-H-CH_2_O]^–^; 297.0409[M-H-C_5_H_10_]^–^; 281.0456[M-H-C_5_H_10_O]^–^; 265.0507[M-H-C_6_H_14_O]^–^; 203.0712[M-H-C_10_H_12_O_2_]^–^	Glycycoumarin	GC
108^a^	23.52	C_21_H_20_O_5_	352.1311	0.7	351.1245[M-H]^–^; 335.0948[M-H-CH_4_]^–^; 321.0815[M-H-CH_2_O]^–^; 307.0973[M-H-C_2_H_4_O]^–^; 293.0810[M-H-C_3_H_6_O]^–^; 281.0488[M-H-C_5_H_10_]^–^; 251.1091[M-H-C_6_H_12_O]^–^; 199.0767[M-H-C_9_H_12_O_2_]^–^	Gancaonin M	GC
109^a*∗*^	23.67	C_21_H_22_O_5_	354.1467	2.5	353.1398[M-H]^–^; 323.0924[M-H-CH_2_O]^–^; 283.0617[M-H-C_5_H_10_]^–^; 269.0443[M-H-C_6_H_12_]^–^; 211.0384[M-H-C_7_H_10_O_3_]^–^; 203.0734[M-H-C_9_H_10_O_2_]^–^	Dehydroglyasperin C	GC
110^a^	23.67	C_21_H_22_O_5_	354.1467	2.5	353.1398[M-H]^–^; 323.0924[M-H-CH_2_O]^–^; 283.0617[M-H-C_5_H_10_]^–^; 269.0443[M-H-C_6_H_12_]^–^; 241.0512[M-H-C_5_H_4_O_3_]^–^	Licochalcone D	GC
111^a*∗*^	24.27	C_25_H_26_O_5_	406.178	0.8	405.1685[M-H]^–^; 311.1714[M-H-C_6_H_6_O]^–^	6,8-Diprenylgenistein	GC
112^a^	24.27	C_20_H_16_O_6_	352.0947	3.8	351.0887[M-H]^–^; 321.0413[M-H-C_2_H_6_]^–^; 307.0981[M-H-C_2_H_4_O]^–^; 283.0983[M-H-C_5_H_8_]^–^; 265.0872[M-H-C_5_H_10_O]^–^; 241.0877[M-H-C_6_H_6_O_2_]^–^; 199.0768[M-H-C_7_H_4_O_4_]^–^	Semilicoisoflavone B	GC
113^e^	24.47	C_22_H_22_O_6_	382.1416	−0.2	381.1356[M-H]^–^; 365.1063[M-H-CH_3_]^–^; 351.0882[M-H-CH_3_O]^–^; 335.0913[M-H-C_2_H_6_O]^–^; 311.0587[M-H-C_5_H_9_]^–^; 307.0978[M-H-C_4_H_8_O]^–^; 295.0981[M-H-C_5_H_10_O]^–^; 283.0975[M-H-C_6_H_12_O]^–^; 255.0313[M-H-C_10_H_12_O]^–^; 241.0875[M-H-C_7_H_8_O_3_]^–^; 217.0528[M-H-C_10_H_12_O_2_]^–^; 201.0200[M-H-C_11_H_14_O_3_]^–^; 175.0391[M-H-C_13_H_16_O_2_]^–^; 161.0249[M-H-C_13_H_16_O_3_]^–^; 135.0098[M-H-C_14_H_16_O_4_]^–^; 107.0508[M-H-C_16_H_17_O_4_]^–^	Glucyrin	GC
114^e*∗*^	24.67	C_21_H_18_O_6_	366.1103	−1	365.1034[M-H]^–^; 335.0567[M-H-CH_2_O]^–^; 295.0251[M-H-C_5_H_10_]^–^; 207.0453[M-H-C_9_H_2_O_3_]^–^	Neoglycyrol	GC
115^a*∗*^	24.69	C_20_H_16_O_5_	336.0997	1.4	335.0928[M-H]^–^; 319.0617[M-H-CH_4_]^–^; 317.0816[M-H-H_2_O]^–^; 305.0454[M-H-C_2_H_6_]^–^; 291.0673[M-H-CO_2_]^–^	Phaseol	GC
116^b^	25.09	C_30_H_46_O_5_	486.3345	−1.7	485.3281[M-H]-; 441.3383[M-H-CO_2_]-; 355.2640[M-H-C_6_H_10_O_3_]-; 303.2334[M-H-C_10_H_14_O_3_]^–^	Echinatic acid	GC
117^b^	25.09	C_30_H_46_O_5_	486.3345	−1.7	485.3281[M-H]-; 441.3010[M-H-C_2_H_4_O]-; 355.2640[M-H-C_6_H_10_O_3_]-; 303.2334[M-H-C_10_H_16_O_3_]^–^	Isoechinatic acid	GC
118^b^	25.09	C_30_H_46_O_5_	486.683	−1.7	485.3281[M-H]-; 441.3383[M-H-CO_2_]-; 355.2640[M-H-C6H_10_O_3_]-; 303.2334[M-H-C_10_H_14_O_3_]^–^	Triphyllic acid	BS
119^a^	25.26	C_20_H_18_O_4_	322.1205	2.3	321.1138[M-H]^–^; 305.0800[M-H-CH_4_]^–^; 291.0663[M-H-C_2_H_6_]^–^; 247.0724[M-H-C_4_H_10_O]^–^; 175.0748[M-H-C_9_H_6_O_2_]^–^; 145.0305[M-H-C_11_H_12_O_2_]^–^; 130.9677[M-H-C_12_H_14_O_2_]^–^	Licoflavone A	BS
120^a*∗*^	25.26	C_20_H_18_O_4_	322.1205	2.3	321.1138[M-H]^–^; 305.0800[M-H-CH_4_]^–^; 291.0663[M-H-C_2_H_6_]^–^; 175.0748[M-H-C_9_H_6_O_2_]^–^; 145.0305[M-H-C_11_H_12_O_2_]^–^; 130.9677[M-H-C_12_H_14_O_2_]^–^; 107.0502[M-H-C_13_H_10_O_3_]^–^	Isobavachromene	GC
121^d^	26.4	C_16_H_22_O_4_	278.1518	−1.5	205.1587[M-H-C_4_H_8_0]^–^; 165.0559[M-H-C_8_H_16_]^–^; 147.0119[M-H-C_8_H_18_O]^–^; 127.1123[M-H-C_9_H_10_O_2_]^–^; 119.0122[M-H-C_9_H_18_O_2_]^–^; 103.0170[M-H-C_9_H_18_O_3_]^–^	Diisobutyl phthalate	GC
122^d^	26.4	C_16_H_22_O_4_	278.1518	−1.5	205.1587[M-H-C_4_H_8_0]^–^; 165.0559[M-H-C_8_H_16_]^–^; 147.0119[M-H-C_8_H_18_O]^–^; 127.1123[M-H-C_9_H_10_O_2_]^–^; 119.0122[M-H-C_9_H_18_O_2_]^–^; 103.0170[M-H-C_9_H_18_O_3_]^–^	Dibutyl phthalate	GC
123^a*∗*^	26.66	C_25_H_28_O_4_	392.1987	−1	391.1933[M-H]^–^; 213.0934[M-H-C_11_H_14_O_2_]^–^; 203.0717[M-H-C_12_H_12_O_2_]^–^; 201.0605[M-H-C_12_H_14_O_2_]^–^; 187.1130[M-H-C_13_H_16_O_2_]^–^; 159.0835[M-H-C_14_H_16_O_3_]^–^; 157.0661[M-H-C_14_H_18_O_3_]^–^	Hispaglabridin A	GC
124^b^	27.78	C_30_H_46_O_4_	470.3396	−1.7	469.3332[M-H]^−^; 425.3438[M-H-CHO_2_]^–^	Liquiritic acid	
125^b*∗*^	28.79	C_30_H_44_O_4_	468.324	−2.6	467.3189[M-H]^−^; 423.3266[M-H-O_2_]^–^	Glabrolide	
126^b^	29.86	C_30_H_48_O_3_	456.3603	−3.1	455.3507[M-H]^–^; 391.2371[M-H-CH_4_O_2_]^–^	(3*β*)-3-hydroxyurs-12-en-30-oic acid	

a: flavonoids, b: triterpenoids, c: monoterpenoid glycosides, d: other components, e: coumarins, *∗* indicates the first discovered component.

**Table 4:** The first 20 key targets of SGD in treating liver injury.

No.	Target name	Betweenness centrality	Closeness centrality	Degree
1	EGFR	0.0461	0.6045	104
2	CTNNB1	0.0527	0.5978	101
3	HSP90AA1	0.0561	0.5833	94
4	SRC	0.0239	0.5821	93
5	HRAS	0.0260	0.5600	86
6	STAT3	0.0176	0.5588	83
7	MAPK1	0.0232	0.5485	75
8	PIK3CA	0.0051	0.5086	59
9	FGF2	0.0047	0.5165	58
10	APP	0.0276	0.5341	56
11	BCL2L1	0.0050	0.5236	54
12	GSK3B	0.0059	0.5175	51
13	KDR	0.0035	0.5135	49
14	NRAS	0.0034	0.4926	49
15	IL2	0.0047	0.5057	49
16	ACE	0.0167	0.5096	47
17	MMP2	0.0020	0.5076	45
18	RELA	0.0067	0.5106	44
19	KIT	0.0033	0.4953	43
20	PRKCA	0.0078	0.5076	43

**Table 5:** The possible binding site (amino acid residue) of the target of the molecular docking results.

Name	Binding energy (kcal/mol)	Hydrogen bonding sites	Hydrogen bond length	Hydrophobic action site
(A) Liquiritin with CTNNB1.	−7.5	Trp338(A), Asn380(A), Arg342(A), Lys345(A)	3.28 Å, 3.07 Å, 3.19 Å, 2.84 Å, 3.12 Å	Lys312(A), Val349(A), Val346(A), Tyr306(A), Gln302(A)
(B) Liquiritin with EGFR.	−8.9	Thr766(A)	2.70 Å	Pro770(A), Lys704(A), Leu768(A), Ala719(A), Lys721(A), Leu764(A), Glu738(A), Thr830(A), Leu820(A), Asp831(A), Met769(A), Gly772(A), Leu694(A)
(C) Liquiritin with MAPK1.	−9.1	Met108(A)	2.80 Å	Leu107(A), Ala52(A), Thr110(A), Asp111(A), Gly32(A), Glu33(A), Lys114(A), Tyr113(A), Ser153(A), Val39(A), Leu156(A), Glu109(A)
(D) Liquiritin with STAT3.	−7.4	Nonexistent	Nonexistent	Lys615(A), Val563(A), Cys468(A), Pro471(A), Met470(A), Arg335(A), Ile569(A), Thr515(A), Lys573(A), Asp570(A), Asn567(A)
(E) Benzoyl paeoniflorin with HRAS.	−10.9	Cys32(X), Asn116(X), Ser17(X), Lys16(X)	2.94 Å, 2.83 Å, 3.13 Å, 3.07 Å	Gln61(X), Asp33(X), Ile21(X), Val29(X), Glu37(X), Glu31(X), Asp30(X), Phe28(X), Lys117(X), Gly15(X), Asp119(X), Ala146(X), Ala18(X), Gly13(X)
(F) Benzoyl paeoniflorin with PIK3CA.	−10.0	Asn756(A), Arg662(A), Cys838(A)	3.12 Å, 2.85 Å, 3.10 Å	Phe666(A), Ser629(A), Ala758(A), Pro757(A), Asn170(A), His670(A), Glu259(A), Glu849(A), Arg818(A), Ile633(A), Gly837(A), Met811(A)
(G) Benzoyl paeoniflorin with SRC.	−10.1	ASer345(A), Thr338(A)	3.12 Å, 2.81 Å	Gly344(A), Leu273(A), Tyr340(A), Leu393(A), Ala293(A), Ala403(A), Val323(A), Phe405(A), Asp404(A), Val281(A), Lys295(A), Ala390(A)
(H) Benzoyl paeoniflorin with HSP90AA1	−10.7	Thr184(A)	2.92 Å	Val150(A), Asp93(A), Met98(A), Val186(A), Trp162(A), Phe138(A), Leu107(A), Leu48(A), Gly135(A), Ile96(A), Ala55(A), Asn51(A), Arg58(A), Ser52(A)

**Table 6:** Calibration curves of the analytes.

Analytes	Calibration curve	Linear rang (ng.*μ*L^−1^)	*r*	LOQ (ng/*μ*L^−1^)
Hesperidin	*Y* = 2*E* + 06*X* + 6462	0.0392∼0.98	0.9992	0.01
Naringenin	*Y* = 2*E* + 06*X* − 3159	0.0384∼0.96	0.9985	0.01
Liquiritin	*Y* = 3*E* + 06*X* + 4*E* + 04	1.432∼35.8	0.9979	0.04
Glycyrrhizic acid	*Y* = 835033*X* + 1*E* + 04	4.62∼115.5	0.9825	1.20
Liquiritigenin	*Y* = 7*E* + 06*X* + 4*E* + 04	0.554∼13.9	0.9990	0.12
Isoliquiritin	*Y* = 4*E* + 06*X* + 20213	0.201∼5.025	0.9998	0.05
Isoliquiritin apioside	*Y* = 2*E* + 06*X* + 50766	0.776∼19.4	1.0000	0.02
Caffeic acid	*Y* = 1*E* + 07*X* + 27935	0.036∼0.09	0.9988	0.01
Ferulic acid	*Y* = 1*E* + 06*X* + 7406	0.006∼0.15	0.9985	0.002
Coniferyl ferulate	*Y* = 625840*X* + 3624	0.008∼0.20	0.9989	0.002
Albiflorin	*Y* = 33658*X* + 44760	2.74∼68.00	0.9978	0.68
Gallic acid	*Y* = 4*E* + 07*X* + 3*E* + 04	0.132∼3.30	0.9997	0.33
Methyl gallate	*Y* = 1*E* + 07*X* + 9012	0.008∼0.20	0.9999	0.002
Benzoyl paeoniflorin	*Y* = 24147*X* − 2883.4	0.42∼10.50	0.9991	0.11
Narirutin	*Y* = 2*E* + 06*X* + 1108	0.008∼0.20	0.9999	0.002
Chlorogenic acid	*Y* = 6*E* + 06*X* + 6980	0.0024∼0.06	0.9990	0.001
Neochlorogenic acid	*Y* = 5*E* + 06*X* + 2745	0.0032∼0.08	0.9995	0.001
Rutin	*Y* = 1*E* + 06*X* + 7917	0.0012∼0.03	0.9994	0.001
1,2,3,4,6-penta-O-galloyl-*β*-D-glucopyranose	*Y* = 1*E* + 06*X* + 3214	0.0010∼0.025	0.9995	0.001

**Table 7:** Contents of 19 components.

Analytes	Contents (g/g)										
	S1	S2	S3	S4	S5	S6	S7	S8	S9	S10	Mean
Hesperidin	0.35	0.32	0.40	0.11	0.24	0.35	0.24	0.26	0.25	0.30	0.28
Naringenin	0.42	0.22	0.51	0.22	0.26	0.31	0.32	0.19	0.26	0.33	0.30
Liquiritin	10.50	11.41	11.57	11.42	12.26	18.91	21.36	13.13	12.77	13.97	13.73
Glycyrrhizic acid	31.33	33.71	31.61	34.04	33.91	45.09	39.31	35.53	31.59	32.46	34.36
Liquiritigenin	1.80	0.58	1.28	0.66	0.59	1.07	1.11	0.88	1.13	1.03	1.01
Isoliquiritin	1.29	1.83	1.28	1.89	1.87	1.61	1.71	1.46	1.33	1.43	1.57
Isoliquiritin apioside	1.49	2.43	1.31	2.52	2.98	3.43	3.62	3.29	1.64	1.68	2.44
Caffeic acid	0.35	0.68	0.67	0.35	0.23	0.44	0.50	0.22	0.31	0.34	0.41
Ferulic acid	0.05	0.12	0.13	0.05	0.06	0.09	0.04	0.05	0.09	0.03	0.07
Coniferyl ferulate	0.08	0.10	0.11	0.07	0.09	1.00	0.09	0.07	0.08	0.09	0.18
Albiflorin	9.97	8.65	9.38	10.32	9.12	7.3	7.51	9.62	8.98	7.87	8.87
Gallic acid	2.11	2.46	2.83	2.71	2.95	2.01	2.10	2.38	2.06	2.40	2.40
Methyl gallate	0.10	0.05	0.09	0.07	0.09	1.01	1.09	0.07	1.08	1.09	0.47
Benzoyl paeoniflorin	2.71	2.09	1.91	1.79	1.91	0.79	0.81	0.80	1.00	0.92	1.47
Narirutin	0.08	0.12	0.07	0.07	0.09	1.01	1.02	1.02	0.08	0.09	0.37
Chlorogenic acid	0.02	0.03	0.03	0.02	0.03	0.02	0.04	0.07	0.02	0.04	0.03
Neochlorogenic acid	0.05	0.07	0.02	0.06	0.06	1.01	0.06	0.04	0.05	0.04	0.15
Rutin	0.02	0.02	0.03	0.03	0.02	0.03	0.02	0.02	0.05	0.04	0.02
1,2,3,4,6-penta-O-galloyl-*β*-D-glucopyranose	0.02	0.03	0.08	0.03	0.04	0.04	0.05	0.05	0.07	0.04	0.05

## Data Availability

The data used to support the findings of this study are available from the corresponding author upon request.

## Supplementary Materials

Supplementary Materials Table 1: Binding energies of representative compounds and targets. Table 2. 128 blood absorbed components. Figure 1: KEGG analysis of potential target genes of SGD, top 20 clusters of KEGG. Figure 2: GO analysis of potential target genes of the SGD.
