# Supplementary material for: Screening of Biomarkers and Quality Control of Shaoyao Gancao Decoction Using UPLC-MS/MS Combined with Network Pharmacology and Molecular Docking Technology
Source: Evid Based Complement Alternat Med. 2022 Nov 29;2022:2442681. doi: 10.1155/2022/2442681 (PMC9726270; doi:10.1155/2022/2442681)
Supplement: Supplementary Materials — Table 1: Binding energies of representative compounds and targets. Table 2. 128 blood absorbed components. Figure 1: KEGG analysis of potential target genes of SGD, top 20 clusters of KEGG. Figure 2: GO analysis of potential target genes of the SGD. [file 2442681.f1.zip › 1106-Table1. 128 Blood Absorbed Components..docx]

**Table 1: Binding energies of representative compounds and targets.**

| Analytes | EGFR | CTNNB1 | HSP90AA1 | SRC | HRAS | STAT3 | MAPK1 | PIK3CA |
| --- | --- | --- | --- | --- | --- | --- | --- | --- |
| benzoylpaeoniflorin | -8.4 | -7.4 | -10.7 | -10.1 | -10.9 | -7.3 | -8.4 | -10 |
| albiflorin | -7.9 | -6.4 | -9.2 | -8.5 | -8.1 | -7.3 | -8.7 | -8.6 |
| liquiritin | -8.9 | -7.5 | -8.7 | -9.7 | -10.2 | -7.4 | -9.1 | -9.3 |
| liquiritigenin | -7.1 | -6.7 | -8.9 | -8.4 | -8.5 | -7.1 | -8.8 | -8.6 |


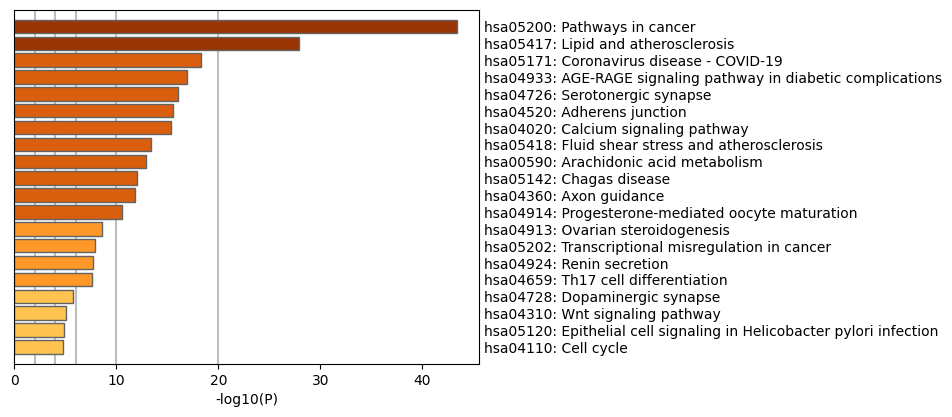


**Figure 1:** KEGG analysis of potential target genes of SGD, top 20 clusters of KEGG.


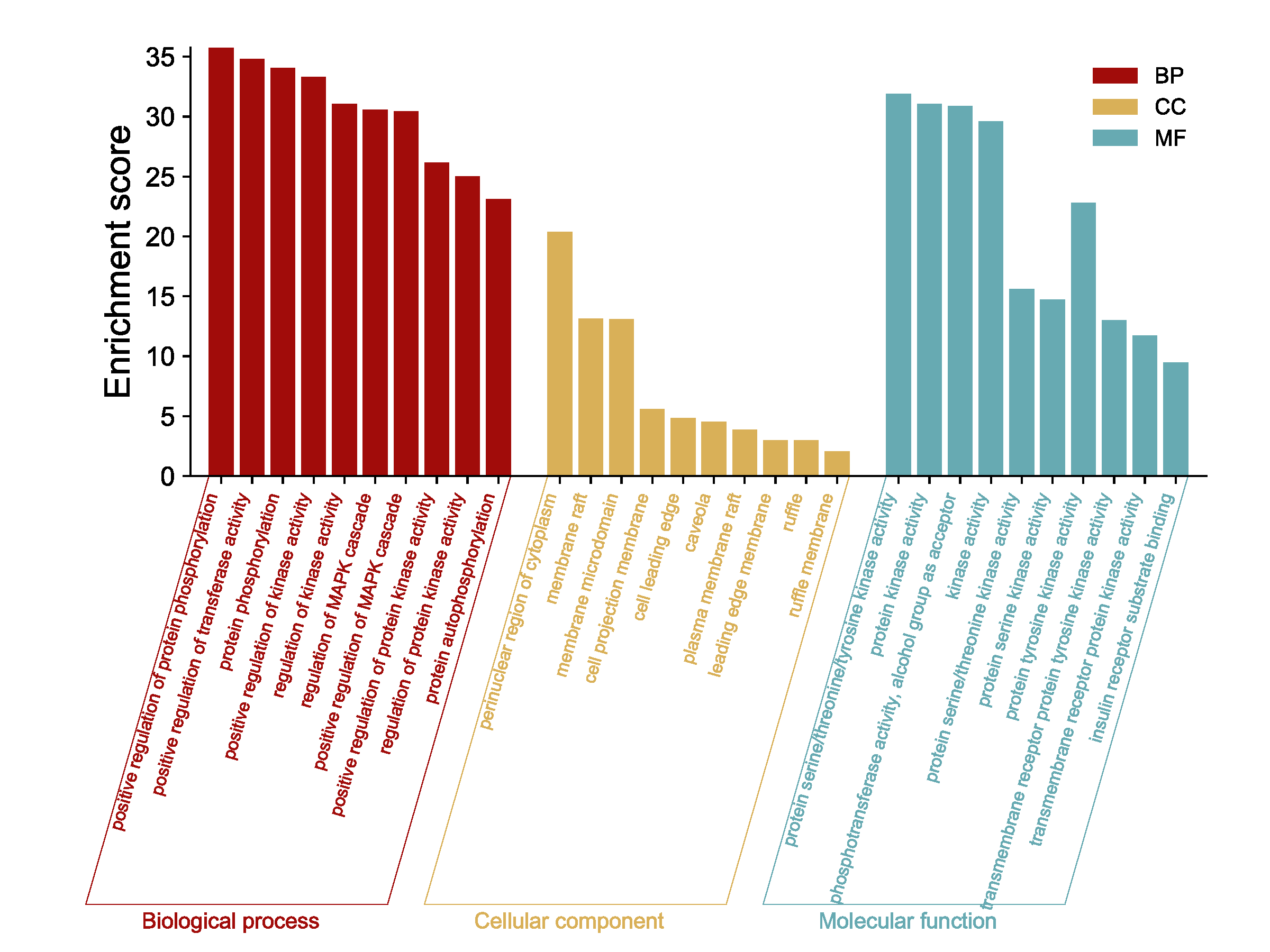


**Figure 2:** GO analysis of potential target genes of the SGD.

Table2. 128 Blood Absorbed Components.

| **No.** | **compound** | **molecular formula** | **Mass**  **（Da）** | **ppm** | **RT（min）** | **MS/MS** | **type** |
| --- | --- | --- | --- | --- | --- | --- | --- |
| 1 | 1: liquiritigenin | C15H12O4 | 257.0808 | 0.5 | 10.23 | 137.0233 C7H5O3+ | P |
| 2 | M1.1 | C21H20O10 | 433.1129 | 0.1 | 14.66 | 257.0812 C15H13O4+ 137.0233 C8H9O2+ | M |
| 3 | M1.2 | C15H12O7S | 337.0377 | 0.2 | 17.29 | 137.0230 C8H9O2+ 257.0821 C15H13O4+ |  |
| 4 | M1.3 | C21H22O9 | 419.1337 | -2.3 | 14 | 257.0813 C15H13O4+ 137.0220 C8H9O2+ |  |
| 5 | M1.4 | C15H14O4 | 259.0965 | 0.9 | 14.2 | 107.0484 C7H7O+ |  |
| 6 | M1.5 | C16H17O4 | 274.1200 | -4.6 | 11.94 | 215.0703 C13H11O3+ 257.0808 C15H13O4+ 231.1016 C14H15O3+ |  |
| 7 | M1.6 | C16H14O4 | 271.0965 | -0.7 | 16.73 | 137.0586 C8H9O2+ |  |
| 8 | 2: isoliquiritigenin | C15H12O4 | 257.0808 | 0.2 | 14.7 | 137.0233 C7H5O3+ | p |
| 9 | M 2.1 | C16H18O4 | 275.1278 | -0.5 | 19.28 | 133.0648 C9H9O+ 105.0699 C8H9+ 275.1278 C16H19O4+ 151.0754 C9H11O2+ 179.1067 C11H15O2+ | M |
| 10 | M2.2 | C15H14O4 | 259.0965 | -0.9 | 15.14 | 121.0648 C8H9O+ |  |
| 11 | M2.3 | C16H14O4 | 271.0965 | -0.7 | 16.73 | 137.0233 C7H5O3+ |  |
| 12 | M2.4 | C16H16O4 | 273.1121 | -0.9 | 15.5 | 137.0233 C7H5O3+ 123.0441 C7H7O2+ 163.0754 C10H11O2+ 103.0542 C8H7+ |  |
| 13 | M2.5 | C15H14O4 | 259.0965 | 0.9 | 14.2 | 107.0491 C7H7O+ |  |
| 14 | M2.6 | C18H18O4 | 299.1278 | 0.6 | 11.41 | 107.0491 C7H7O+ 191.0703 C11H11O3+ 145.0284 C9H5O2+ 251.0703 C16H11O3+ |  |
| 15 | 3: hesperidin | C28H34O15 | 609.1834 | 1.6 | 12.58 | 609.1834 C28H33O15- | p |
| 16 | M3.1 | C28H34O15 | 609.1834 | 1.6 | 12.58 | 609.1834 C28H33O15- | M |
| 17 | 4: naringenin | C15H12O5 | 273.0758 | 0.1 | 12.48 | 153.0185 C7H5O4+ | P |
| 18 | M4.1 | C7H4O4 | 153.0185 | 0.1 | 14.27 | 153.0185 C7H5O4+ | M |
| 19 | 5: liquiritin | C21H22O9 | 419.1337 | -0.2 | 14.02 | 257.0808 C15H13H4+ 137.0233 C7H5O3+ | p |
| 20 | M5.1 | C15H12O4 | 257.0810 | 10.23 | 0.5 | 137.0233 C7H5O3+ 147.0441 C9H7O2+ | M |
| 21 | M5.2 | C15H12O5 | 273.0755 | 12.5 | -1.1 | 153.0546 C8H9O3+ |  |
| 22 | M5.3 | C15H12O7S | 337.0377 | 17.29 | 0.2 | 137.0233 C7H5O3+ 257.0808 C15H13O4+ |  |
| 23 | M5.4 | C16H18O4 | 275.1277 | 19.28 | -0.5 | 133.0648 C9H9O+ 105.0699 C8H9+ 275.1278 C16H19O4+ 151.0754 C9H11O2+ 179.1067 C11H15O2+ |  |
| 24 | M5.5 | C21H20O10 | 433.1130 | 14.66 | 0.1 | 257.0808 C15H13O4+ 137.0233 C7H5O3+ |  |
| 25 | M5.6 | C21H20O11 | 449.1078 | 12.5 | -0.2 | 273.0758 C15H13O5+ 153.0546 C8H9O3+ |  |
| 26 | M5.7 | C16H14O5 | 287.0910 | 12.05 | -1.4 | 123.0441 C7H7O2+ |  |
| 27 | M5.8 | C16H14O4 | 271.0963 | 16.73 | -0.7 | 137.0597 C8H9O2+ 109.0648 C7H9O+ |  |
| 28 | 6: glycyrrhizic acid | C42H62O16 | 821.4001 | 21.23 | 4.4 | 821.4040 C42H61O16- | P |
| 29 | M6.1 | C42H62O16 | 821.4001 | 21.23 | 4.4 | 821.4040 C42H61O16- | M |
| 30 | 7: isoliquiritin | C_21_H_22_O_9_ | 419.1337 | 14.00 | -2.3 | 257.0808 C_15_H_13_O_4_^+^ 137.0233 C_7_H_5_O_3_^+^ | P |
| 31 | M7.1 | C15H12O4 | 257.0810 | 0.5 | 10.23 | 137.0233 C7H5O3+ 147.0441 C9H7O2+ | M |
| 32 | M7.2 | C15H12O7S | 337.0377 | 0.2 | 17.29 | 137.0233 C7H5O3+ 257.0808 C15H13O4+ |  |
| 33 | M7.3 | C16H18O4 | 275.1277 | -0.5 | 19.28 | 133.0648 C9H9O+ 105.0699 C8H9+ 275.1278 C16H19O4+ 151.0754 C9H11O2+ |  |
| 34 | M7.4 | C21H20O10 | 433.1130 | 0.1 | 14.66 | 257.0808 C15H13O4+ 137.0233 C7H5O3+ |  |
| 35 | M7.5 | C16H14O4 | 271.0963 | -0.7 | 16.73 | 137.0233 C7H5O3+ 109.0284 C6H5O2+ |  |
| 36 | M7.6 | C16H16O4 | 273.1119 | -0.9 | 15.5 | 137.0233 C7H5O3+ 123.0441 C7H7O2+ 163.0754 C10H11O2+ 103.0542 C8H7+ |  |
| 37 | M7.7 | C15H14O7S | 339.0532 | -0.3 | 13.74 | 107.0491 C7H7O+ |  |
| 38 | M7.8 | C21H22O10 | 435.1290 | 0.9 | 14.2 | 107.0491 C7H7O+ |  |
| 39 | M7.9 | C21H22O10 | 435.1290 | 0.9 | 14.2 | 107.0703 C4H11O3+ |  |
| 40 | 8: isoliquiritin apioside | C26H30O13 | 549.1616 | 0.5 | 10.11 | 255.0674 C15H11O4- | P |
| 41 | M8.1 | C_15_H_11_O_4_ | 255.0674 | -0.9 | 10.23 | 255.0674 C15H11O4- | M |
| 42 | 9: caffeic acid | C9H8O4 | 179.0348 | -0.8 | 13.01 | 135.0444 C8H7O2- | P |
| 43 | M9.1 | C_8_H_7_O_2_ | 134.0374 | 0.4 | 26.41 | 135.0444 C8H7O2- | M |
| 44 | 10: ferulic acid | C10H10O4 | 193.0503 | -1.5 | 6.22 | 133.0279 C8H5O2- | P |
| 45 | M10.1 | C8H6O2 | 133.0290 | -4.5 | 16.06 | 133.0279 C8H5O2- | M |
| 46 | 11: coniferyl ferulate | C20H20O6 | 357.1338 | 1.5 | 17.96 | 175.0373 C10H7O3- | P |
| 47 | M11.1 | C_10_H_8_O_3_ | 175.0402 | 0.5 | 5.91 | 175.0373 C10H7O3- | M |
| 48 | 12: albiflorin | C23H28O11 | 479.1588 | -2.9 | 24.68 | 479.15877 C23H27O11- | P |
| 49 | M12.1 | C_23_H_27_O_11_ | 479.1588 | -2.9 | 24.68 | 479.15877 C23H27O11- | M |
| 50 | 13: gallic acid | C7H6O5 | 125.0237 | 2.9 | 3.28 | 122.8936 C6H3O3- | P |
| 51 | M13.1 | C6H4O3 | 123.0082 | -5 | 32.15 | 122.8936 C6H3O3- | M |
| 52 | 14: methyl gallate | C8H8O5 | 183.0290 | -4.9 | 0.97 | 183.0290 C8H7O5- | P |
| 53 | M14.1 | C8H8O5 | 183.0290 | -4.9 | 0.97 | 183.0290 C8H7O5- | M |
| 54 | 15: benzoyl paeoniflorin | C30H32O12 | 583.1828 | 1.2 | 16.88 | 195.0680 C10H11O4- | P |
| 55 | M15.1 | C10H12O4 | 195.0653 | -5.3 | 8.69 | 139.1481 C10H19+ 111.1168 C8H15+ 125.1325 C9H17+ | M |
| 56 | 16: narirutin | C27H32O14 | 579.1739 | 3.3 | 21.01 | 511.1882 C23H27O13- | P |
| 57 | M16.1 | C_23_H_28_O_13_ | 579.1739 | 3.3 | 21.01 | 511.1882 C23H27O13- | M |
| 58 | 17: chlorogenic acid | C16H18O9 | 309.0969 | 2.4 | 15.08 | 353.2121 C16H17O9- | P |
| 59 | M17.1 | C15H17O7 | 309.0977 | 0.7 | 15.08 | 353.2121 C16H17O9- | M |
| 60 | 18: neochlorogenic acid | C16H18O9 | 309.0969 | 2.4 | 15.08 | 353.2121 C16H17O9- | P |
| 61 | M18.1 | C15H17O7 | 309.0977 | 0.7 | 15.08 | 353.2121 C16H17O9- | M |
| 62 | 19: rutin | C27H30O16 | 609.1470 | 1.5 | 9.47 | 285.0416 C15H9O6- | P |
| 63 | M19.1 | C15H10O6 | 285.0413 | 2.8 | 9.9 | 285.0416 C15H9O6- | M |
| 64 | 20: 1,2,3,4,6-penta-O-galloyl-β-D-glucopyranose | C41H32O26 | 941.1210 | -4.8 | 14.66 | 941.1210 C41H33O26+ | P |
| 65 | M20.1 | C41H32O26 | 941.1210 | -4.8 | 14.66 | 941.1210 C41H33O26+ | M |
| 66 | 21: vitexin | C21H20O10 | 433.1129 | 0.7 | 14.69 | 137.0597 C8H9O2+ | P |
| 67 | M21.1 | C15H10O5 | 271.0601 | 1.2 | 17.08 | 271.0601 C15H11O5+ | M |
| 68 | M21.2 | C16H12O5 | 285.0758 | -1 | 11.96 | 137.0233 C7H5O3+ |  |
| 69 | M21.3 | C15H12O5 | 273.0758 | -1.1 | 12.5 | 153.0182 C7H5O4+ |  |
| 70 | M21.4 | C15H12O5 | 273.0758 | -1.1 | 12.5 | 153.0182 C7H5O4+ |  |
| 71 | M21.5 | C15H12O5 | 273.0758 | -1.1 | 12.5 | 153.0182 C7H5O4+ |  |
| 72 | M21.6 | C22H22O11 | 463.1235 | 0.6 | 12.85 | 287.0761 C12H15O8+ 121.0495 C4H9O4+ 193.0343 C6H9O7+ |  |
| 73 | M21.7 | C21H20O11 | 449.1078 | -0.2 | 12.5 | 153.0758 C_5_H_13_O_5_+ 147.0441 C_9_H_7_O_2_+ |  |
| 74 | M21.8 | C21H20O11 | 449.1078 | -0.2 | 12.5 | 273.0969 C_12_H_17_O_7_+ 153.0546 C_8_H_9_O_3_+ |  |
| 75 | M21.9 | C22H22O10 | 447.1286 | -1.5 | 16.73 | 137.0808 C15H13O4+ |  |
| 76 | 22: glycyrrhetinic acid | C30H46O4 | 471.3469 | 0.5 | 27.78 | 471.3469 C30H47O4+ | P |
| 77 | M22.1 | C36H54O10 | 647.3790 | -0.7 | 21.2 | 453.3363 C30H45O3+ | M |
| 78 | M22.2 | C42H62O16 | 823.4111 | 0.9 | 21.22 | 453.3363 C30H45O3+ |  |
| 79 | M22.3 | C38H56O11 | 689.3895 | 2.5 | 21.63 | 689.3895 C38H57O11+ 345.1544 C16H25O8+ |  |
| 80 | M22.4 | C35H56O10 | 637.3946 | 4 | 32.52 | 637.3946 C35H57O10+ |  |
| 81 | M22.5 | C34H55NO6 | 574.4102 | 3 | 31.48 | 574.4102 C34H56NO6+ 455.3884 C31H51O2+ |  |
| 82 | M22.6 | C31H48O4 | 485.3625 | 0.5 | 30.71 | 485.3625 C31H49O4+ 331.2268 C21H31O3+ |  |
| 83 | M22.7 | C32H53NO5 | 532.3997 | 1 | 24.25 | 532.3997 C32H54NO5+ 156.0655 C7H10NO3+ 170.0812 C8H12NO3+ 211.2056 C14H27O+ 532.3997 C32H54NO5+ |  |
| 84 | 23: macedonic acid | C30H46O4 | 471.3469 | 0.2 | 21.22 | 471.3469 C30H47O4+ | P |
| 85 | M23.1 | C36H54O10 | 647.3790 | -0.7 | 21.2 | 453.3363 C30H45O3+ 435.3258 C30H43O2+ | M |
| 86 | M23.2 | C36H56O10 | 649.3946 | -0.1 | 21 | 133.0495 C5H9O4+ 177.0394 C6H9O6+ |  |
| 87 | M23.3 | C29H44O4 | 457.3312 | -0.7 | 25.73 | 411.3258 C28H43O2+ 187.1329 C10H19O3+ |  |
| 88 | M23.4 | C30H46O4 | 471.3469 | -1 | 27.77 | 471.3469 C30H47O4+ 317.2111 C20H29O3+ |  |
| 89 | M23.5 | C35H52O10 | 633.3633 | -1.8 | 29.44 | 457.3312 C29H45O4+ 633.3633 C35H53O10+ |  |
| 90 | M23.6 | C31H48O4 | 485.3625 | 0.5 | 30.71 | 485.3625 C31H49O4+ 331.2268 C21H31O3+ |  |
| 91 | M23.7 | C31H48O4 | 485.3625 | 0.5 | 30.71 | 485.3625 C31H49O4+ 331.2268 C21H31O3+ |  |
| 92 | M23.8 | C29H46O4 | 459.3469 | -0.5 | 28.55 | 423.3258 C29H43O2+ 459.3469 C29H47O4+ 157.0859 C8H15O3+ 171.1016 C9H15O3+ |  |
| 93 | M23.9 | C36H56O10 | 649.3946 | -0.1 | 21 | 133.0495 C5H9O4+ 177.0394 C6H9O6+ |  |
| 94 | 24: glycycoumarin | C21H20O6 | 369.1333 | 0.3 | 21.09 | 313.0707 C17H13O6+ 271.0601 C15H11O5+ | P |
| 95 | M24.1 | C21H26O7 | 391.1751 | 2.9 | 29.76 | 149.0233 C8H5O3+ 123.0441 C7H7O2+ 139.0390 C7H7O3+ 391.1751 C21H27O7+ | M |
| 96 | M24.2 | C22H28O7 | 405.1908 | -0.9 | 1.24 | 301.1071 C17H17O5+ |  |
| 97 | M24.3 | C22H22O6 | 383.1489 | 0.4 | 24.45 | 327.0863 C18H15O6+ |  |
| 98 | M24.4 | C33H36O18 | 721.1974 | 1.1 | 15.88 | 313.0918 C14H17O8+ |  |
| 99 | M24.5 | C24H26O6 | 411.1802 | 2.6 | 9.44 | 411.1802 C24H27O6+ |  |
| 100 | 25: glycyrrhetinic acid 3-O-mono-beta-D-glucuronide | C36H54O10 | 647.3790 | 0.9 | 21.22 | 453.3363 C30H45O3+ | P |
| 101 | M25.1 | C30H46O4 | 471.34690 | -1 | 27.77 | 471.3469 C30H47O4+ 317.2111 C20H29O3+ 235.1693 C15H23O2+ | M |
| 102 | M25.2 | C36H54O10 | 647.3790 | -0.7 | 21.2 | 453.3363 C30H45O3+ 435.3258 C30H43O2+ |  |
| 103 | M25.3 | C42H62O16 | 823.4111 | 0.9 | 21.22 | 453.3363 C30H45O3+ 647.3790 C36H55O10+ |  |
| 104 | M25.4 | C36H56O10 | 649.3946 | -0.1 | 21 | 133.0495 C5H9O4+ 177.0394 C6H9O6+ |  |
| 105 | M25.5 | C36H56O10 | 649.3946 | -0.1 | 21 | 133.0495 C5H9O4+ 177.0394 C6H9O6+ |  |
| 106 | 26: retrochalcone | C16H14O4 | 271.0965 | 0 | 16.74 | 137.0597 C8H9O2+ | P |
| 107 | M26.1 | C16H18O4 | 275.1278 | -0.5 | 19.28 | 133.0648 C9H9O+ 105.0335 C7H5O+ 275.1278 C16H19O4+ 151.0754 C9H11O2+ 179.1067 C11H15O2+ | M |
| 108 | M26.2 | C16H16O4 | 273.1121 | -0.9 | 15.5 | 137.0597 C8H9O2+ 123.0441 C7H7O2+ 163.0754 C10H11O2+ |  |
| 109 | M26.3 | C16H16O4 | 273.1121 | -0.9 | 15.5 | 137.0597 C8H9O2+ 123.0441 C7H7O2+ 163.0754 C10H11O2+ 103.0178 C7H3O+ |  |
| 110 | M26.4 | C30H42O16 | 659.2546 | -1.3 | 28.82 | 479.1548 C23H27O11+ |  |
| 111 | M26.5 | C18H20O4 | 301.1434 | -4.8 | 28.09 | 301.1434 C18H21O4+ |  |
| 112 | M26.6 | C18H20O4 | 301.1434 | -4.8 | 28.09 | 301.1434 C18H21O4+ |  |
| 113 | M26.7 | C23H30O10 | 467.1912 | -4.9 | 7.99 | 125.0597 C7H9O2+ |  |
| 114 | 27: licoarylcoumarin | C21H20O6 | 369.1333 | 0.3 | 21.09 | 313.1071 C18H17O5+ 271.0601 C15H11O5+ | P |
| 115 | M27.1 | C21H26O10S | 471.1320 | -2.8 | 23.62 | 171.1380 C10H19O2+ | M |
| 116 | M27.2 | C21H26O7 | 391.1751 | 2.9 | 29.76 | 149.0233 C8H5O3+ 123.0441 C7H7O2+ 139.0390 C7H7O3+ 391.1751 C21H27O7+ |  |
| 117 | M27.3 | C22H22O6 | 383.1489 | 0.4 | 24.45 | 327.1227 C19H19O5+ |  |
| 118 | 28: 4H-1-Benzopyran-4-one,3-[4,6-dihydroxy-2-methoxy-3-(3-methyl-2-buten-1-yl)phenyl]-7-hydroxy- | C21H20O6 | 369.1333 | 0.3 | 21.09 | 313.0707 C17H13O6+ 271.0601 C15H11O5+ | P |
| 119 | M28.1 | C22H22O6 | 383.1489 | 0.4 | 24.45 | 327.0863 C18H15O6+ | M |
| 120 | M28.2 | C21H28O6 | 377.1959 | 0.3 | 25.51 | 197.0808 C10H13O4+ 235.1329 C14H19O3+ 123.0441 C7H7O2+ |  |
| 121 | M28.3 | C21H28O6 | 377.1959 | 0.3 | 25.51 | 197.0808 C10H13O4+ 235.1329 C14H19O3+ 123.0441 C7H7O2+ |  |
| 122 | M28.4 | C24H26O6 | 411.1802 | 2.6 | 9.44 | 411.1802 C24H27O6+ |  |
| 123 | 29: tetradecanoic acid | C14H28O2 | 229.2162 | -0.5 | 30.61 | 117.0910 C6H13O2+ | P |
| 124 | M29.1 | C14H26O4 | 259.1904 | 0.6 | 27.6 | 101.0597 C5H9O2+ 111.0804 C7H11O+ 129.0910 C7H13O2+ | M |
| 125 | M29.2 | C14H28O6S | 325.1679 | 4.1 | 25.86 | 139.1117 C9H15O+ 125.0961 C8H13O+ |  |
| 126 | M29.3 | C14H28O6S | 325.1679 | 4.1 | 25.86 | 139.1117 C9H15O+ 125.0961 C8H13O+ |  |
| 127 | M29.4 | C14H28O9S2 | 405.1248 | -1.5 | 1.21 | 153.1274 C10H17O+ 211.2056 C14H27O+ |  |
| 128 | M29.5 | C14H28O9S2 | 405.1248 | -1.5 | 1.21 | 153.1274 C10H17O+ 211.2056 C14H27O+ |  |

P stands for Prototype compound, M stands for metabolite.
