# Supplementary material for: Screening of Biomarkers and Quality Control of Shaoyao Gancao Decoction Using UPLC-MS/MS Combined with Network Pharmacology and Molecular Docking Technology
Source: Evid Based Complement Alternat Med. 2022 Nov 29;2022:2442681. doi: 10.1155/2022/2442681 (PMC9726270; doi:10.1155/2022/2442681)
Supplement: Supplementary Materials — Table 1: Binding energies of representative compounds and targets. Table 2. 128 blood absorbed components. Figure 1: KEGG analysis of potential target genes of SGD, top 20 clusters of KEGG. Figure 2: GO analysis of potential target genes of the SGD. [file 2442681.f1.zip › Supplemental Table S1.pdf]

Analytes	EGFR	CTNNB1	HSP90AA1	SRC	HRAS	STAT3	MAPK1	PIK3CA
benzoylpaeoniflorin	-8.4	-7.4	-10.7	-10.1	-10.9	-7.3	-8.4	-10
albiflorin	-7.9	-6.4	-9.2	-8.5	-8.1	-7.3	-8.7	-8.6
liquiritin	-8.9	-7.5	-8.7	-9.7	-10.2	-7.4	-9.1	-9.3
liquiritigenin	-7.1	-6.7	-8.9	-8.4	-8.5	-7.1	-8.8	-8.6
