# Supplementary material for: Screening of Biomarkers and Quality Control of Shaoyao Gancao Decoction Using UPLC-MS/MS Combined with Network Pharmacology and Molecular Docking Technology
Source: Evid Based Complement Alternat Med. 2022 Nov 29;2022:2442681. doi: 10.1155/2022/2442681 (PMC9726270; doi:10.1155/2022/2442681)
Supplement: Supplementary Materials — Table 1: Binding energies of representative compounds and targets. Table 2. 128 blood absorbed components. Figure 1: KEGG analysis of potential target genes of SGD, top 20 clusters of KEGG. Figure 2: GO analysis of potential target genes of the SGD. [file 2442681.f1.zip › Supplemental Table S2.pdf]

No.	compound	molecular formula	Mass （Da）	ppm	RT（min）	MS/MS	type
1	1: liquiritigenin	$C_{15}H_{12}O_4$	257.0808	0.5	10.23	137.0233 $C_7H_5O_3^+$	P
2	M1.1	$C_{21}H_{20}O_{10}$	433.1129	0.1	14.66	257.0812 $C_{15}H_{13}O_4^+$ 137.0233 $C_8H_9O_2^+$	M
3	M1.2	$C_{15}H_{12}O_7S$	337.0377	0.2	17.29	137.0230 $C_8H_9O_2^+$ 257.0821 $C_{15}H_{13}O_4^+$	
4	M1.3	$C_{21}H_{22}O_9$	419.1337	-2.3	14	257.0813 $C_{15}H_{13}O_4^+$ 137.0220 $C_8H_9O_2^+$	
5	M1.4	$C_{15}H_{14}O_4$	259.0965	0.9	14.2	107.0484 $C_7H_7O^+$	
6	M1.5	$C_{16}H_{17}O_4$	274.1200	-4.6	11.94	215.0703 $C_{13}H_{11}O_3^+$ 257.0808 $C_{15}H_{13}O_4^+$ 231.1016 $C_{14}H_{15}O_3^+$	
7	M1.6	$C_{16}H_{14}O_4$	271.0965	-0.7	16.73	137.0586 $C_8H_9O_2^+$	
8	2: isoliquiritigenin	$C_{15}H_{12}O_4$	257.0808	0.2	14.7	137.0233 $C_7H_5O_3^+$	p
9	M 2.1	$C_{16}H_{18}O_4$	275.1278	-0.5	19.28	133.0648 $C_9H_9O^+$ 105.0699 $C_8H_9^+$ 275.1278 $C_{16}H_{19}O_4^+$ 151.0754 $C_9H_{11}O_2^+$ 179.1067 $C_{11}H_{15}O_2^+$	M
10	M2.2	$C_{15}H_{14}O_4$	259.0965	-0.9	15.14	121.0648 $C_8H_9O^+$	
11	M2.3	$C_{16}H_{14}O_4$	271.0965	-0.7	16.73	137.0233 $C_7H_5O_3^+$	
12	M2.4	$C_{16}H_{16}O_4$	273.1121	-0.9	15.5	137.0233 $C_7H_5O_3^+$ 123.0441 $C_7H_7O_2^+$ 163.0754 $C_{10}H_{11}O_2^+$ 103.0542 $C_8H_7^+$	
13	M2.5	$C_{15}H_{14}O_4$	259.0965	0.9	14.2	107.0491 $C_7H_7O^+$	
14	M2.6	$C_{18}H_{18}O_4$	299.1278	0.6	11.41	107.0491 $C_7H_7O^+$ 191.0703 $C_{11}H_{11}O_3^+$ 145.0284 $C_9H_5O_2^+$ 251.0703 $C_{16}H_{11}O_3^+$	
15	3: hesperidin	$C_{28}H_{34}O_{15}$	609.1834	1.6	12.58	609.1834 $C_{28}H_{33}O_{15}^-$	p
16	M3.1	$C_{28}H_{34}O_{15}$	609.1834	1.6	12.58	609.1834 $C_{28}H_{33}O_{15}^-$	M
17	4: naringenin	$C_{15}H_{12}O_5$	273.0758	0.1	12.48	153.0185 $C_7H_5O_4^+$	P
18	M4.1	$C_7H_4O_4$	153.0185	0.1	14.27	153.0185 $C_7H_5O_4^+$	M
19	5: liquiritin	$C_{21}H_{22}O_9$	419.1337	-0.2	14.02	257.0808 $C_{15}H_{13}H_4^+$ 137.0233 $C_7H_5O_3^+$	p
20	M5.1	$C_{15}H_{12}O_4$	257.0810	10.23	0.5	137.0233 $C_7H_5O_3^+$ 147.0441 $C_9H_7O_2^+$	M
21	M5.2	$C_{15}H_{12}O_5$	273.0755	12.5	-1.1	153.0546 $C_8H_9O_3^+$	
22	M5.3	$C_{15}H_{12}O_7S$	337.0377	17.29	0.2	137.0233 $C_7H_5O_3^+$ 257.0808 $C_{15}H_{13}O_4^+$	
23	M5.4	$C_{16}H_{18}O_4$	275.1277	19.28	-0.5	133.0648 $C_9H_9O^+$ 105.0699 $C_8H_9^+$ 275.1278 $C_{16}H_{19}O_4^+$ 151.0754 $C_9H_{11}O_2^+$ 179.1067 $C_{11}H_{15}O_2^+$	
24	M5.5	$C_{21}H_{2}0O_{10}$	433.1130	14.66	0.1	257.0808 $C_{15}H_{13}O_4^+$ 137.0233 $C_7H_5O_3^+$	
25	M5.6	$C_{21}H_{20}O_{11}$	449.1078	12.5	-0.2	273.0758 $C_{15}H_{13}O_5^+$ 153.0546 $C_8H_9O_3^+$	

26	M5.7	$C_{16}H_{14}O_5$	287.0910	12.05	-1.4	123.0441 $C_7H_7O_2^+$	
27	M5.8	$C_{16}H_{14}O_4$	271.0963	16.73	-0.7	137.0597 $C_8H_9O_2^+$ 109.0648 $C_7H_9O^+$	
28	6: glycyrrhizic acid	$C_{42}H_{62}O_{16}$	821.4001	21.23	4.4	821.4040 $C_{42}H_{61}O_{16}^-$	P
29	M6.1	$C_{42}H_{62}O_{16}$	821.4001	21.23	4.4	821.4040 $C_{42}H_{61}O_{16}^-$	M
30	7: isoliquiritin	$C_{21}H_{22}O_9$	419.1337	14.00	-2.3	257.0808 $C_{15}H_{13}O_4^+$ 137.0233 $C_7H_5O_3^+$	P
31	M7.1	$C_{15}H_{12}O_4$	257.0810	0.5	10.23	137.0233 $C_7H_5O_3^+$ 147.0441 $C_9H_7O_2^+$	
32	M7.2	$C_{15}H_{12}O_7S$	337.0377	0.2	17.29	137.0233 $C_7H_5O_3^+$ 257.0808 $C_{15}H_{13}O_4^+$	
33	M7.3	$C_{16}H_{18}O_4$	275.1277	-0.5	19.28	133.0648 $C_9H_9O^+$ 105.0699 $C_8H_9^+$ 275.1278 $C_{16}H_{19}O_4^+$ 151.0754 $C_9H_{11}O_2^+$	
34	M7.4	$C_{21}H_{20}O_{10}$	433.1130	0.1	14.66	257.0808 $C_{15}H_{13}O_4^+$ 137.0233 $C_7H_5O_3^+$	
35	M7.5	$C_{16}H_{14}O_4$	271.0963	-0.7	16.73	137.0233 $C_7H_5O_3^+$ 109.0284 $C_6H_5O_2^+$	M
36	M7.6	$C_{16}H_{16}O_4$	273.1119	-0.9	15.5	137.0233 $C_7H_5O_3^+$ 123.0441 $C_7H_7O_2^+$ 163.0754 $C_{10}H_{11}O_2^+$ 103.0542 $C_8H_7^+$	
37	M7.7	$C_{15}H_{14}O_7S$	339.0532	-0.3	13.74	107.0491 $C_7H_7O^+$	
38	M7.8	$C_{21}H_{22}O_{10}$	435.1290	0.9	14.2	107.0491 $C_7H_7O^+$	
39	M7.9	$C_{21}H_{22}O_{10}$	435.1290	0.9	14.2	107.0703 $C_4H_{11}O_3^+$	
40	8: isoliquiritin apioside	$C_{26}H_{30}O_{13}$	549.1616	0.5	10.11	255.0674 $C_{15}H_{11}O_4^-$	P
41	M8.1	$C_{15}H_{11}O_4$	255.0674	-0.9	10.23	255.0674 $C_{15}H_{11}O_4^-$	M
42	9: caffeic acid	$C_9H_8O_4$	179.0348	-0.8	13.01	135.0444 $C_8H_7O_2^-$	P
43	M9.1	$C_8H_7O_2$	134.0374	0.4	26.41	135.0444 $C_8H_7O_2^-$	M
44	10: ferulic acid	$C_{10}H_{10}O_4$	193.0503	-1.5	6.22	133.0279 $C_8H_5O_2^-$	P
45	M10.1	$C_8H_6O_2$	133.0290	-4.5	16.06	133.0279 $C_8H_5O_2^-$	M
46	11: coniferyl ferulate	$C_{20}H_{20}O_6$	357.1338	1.5	17.96	175.0373 $C_{10}H_7O_3^-$	P
47	M11.1	$C_{10}H_8O_3$	175.0402	0.5	5.91	175.0373 $C_{10}H_7O_3^-$	M
48	12: albiflorin	$C_{23}H_{28}O_{11}$	479.1588	-2.9	24.68	479.15877 $C_{23}H_{27}O_{11}^-$	P
49	M12.1	$C_{23}H_{27}O_{11}$	479.1588	-2.9	24.68	479.15877 $C_{23}H_{27}O_{11}^-$	M
50	13: gallic acid	$C_7H_6O_5$	125.0237	2.9	3.28	122.8936 $C_6H_3O_3^-$	P
51	M13.1	$C_6H_4O_3$	123.0082	-5	32.15	122.8936 $C_6H_3O_3^-$	M
52	14: methyl gallate	$C_8H_8O_5$	183.0290	-4.9	0.97	183.0290 $C_8H_7O_5^-$	P
53	M14.1	$C_8H_8O_5$	183.0290	-4.9	0.97	183.0290 $C_8H_7O_5^-$	M
54	15: benzoyl paeoniflorin	$C_{30}H_{32}O_{12}$	583.1828	1.2	16.88	195.0680 $C_{10}H_{11}O_4^-$	P

55	M15.1	$C_{10}H_{12}O_4$	195.0653	-5.3	8.69	139.1481 $C_{10}H_{19}^+$ 111.1168 $C_8H_{15}^+$ 125.1325 $C_9H_{17}^+$	M
56	16: narirutin	$C_{27}H_{32}O_{14}$	579.1739	3.3	21.01	511.1882 $C_{23}H_{27}O_{13}^-$	P
57	M16.1	$C_{23}H_{28}O_{13}$	579.1739	3.3	21.01	511.1882 $C_{23}H_{27}O_{13}^-$	M
58	17: chlorogenic acid	$C_{16}H_{18}O_9$	309.0969	2.4	15.08	353.2121 $C_{16}H_{17}O_9^-$	P
59	M17.1	$C_{15}H_{17}O_7$	309.0977	0.7	15.08	353.2121 $C_{16}H_{17}O_9^-$	M
60	18: neochlorogenic acid	$C_{16}H_{18}O_9$	309.0969	2.4	15.08	353.2121 $C_{16}H_{17}O_9^-$	P
61	M18.1	$C_{15}H_{17}O_7$	309.0977	0.7	15.08	353.2121 $C_{16}H_{17}O_9^-$	M
62	19: rutin	$C_{27}H_{30}O_{16}$	609.1470	1.5	9.47	285.0416 $C_{15}H_9O_6^-$	P
63	M19.1	$C_{15}H_{10}O_6$	285.0413	2.8	9.9	285.0416 $C_{15}H_9O_6^-$	M
64	20: 1,2,3,4,6-penta-O-galloyl-*β*-D-glucopyranose	$C_{41}H_{32}O_{26}$	941.1210	-4.8	14.66	941.1210 $C_{41}H_{33}O_{26}^+$	P
65	M20.1	$C_{41}H_{32}O_{26}$	941.1210	-4.8	14.66	941.1210 $C_{41}H_{33}O_{26}^+$	M
66	21: vitexin	$C_{21}H_{20}O_{10}$	433.1129	0.7	14.69	137.0597 $C_8H_9O_2^+$	P
67	M21.1	$C_{15}H_{10}O_5$	271.0601	1.2	17.08	271.0601 $C_{15}H_{11}O_5^+$	
68	M21.2	$C_{16}H_{12}O_5$	285.0758	-1	11.96	137.0233 $C_7H_5O_3^+$	
69	M21.3	$C_{15}H_{12}O_5$	273.0758	-1.1	12.5	153.0182 $C_7H_5O_4^+$	
70	M21.4	$C_{15}H_{12}O_5$	273.0758	-1.1	12.5	153.0182 $C_7H_5O_4^+$	
71	M21.5	$C_{15}H_{12}O_5$	273.0758	-1.1	12.5	153.0182 $C_7H_5O_4^+$	M
72	M21.6	$C_{22}H_{22}O_{11}$	463.1235	0.6	12.85	287.0761 $C_{12}H_{15}O_8^+$ 121.0495 $C_4H_9O_4^+$ 193.0343 $C_6H_9O_7^+$	
73	M21.7	$C_{21}H_{20}O_{11}$	449.1078	-0.2	12.5	153.0758 $C_5H_{13}O_5$+ 147.0441 $C_9H_7O_2$+	
74	M21.8	$C_{21}H_{20}O_{11}$	449.1078	-0.2	12.5	273.0969 $C_{12}H_{17}O_7$+ 153.0546 $C_8H_9O_3$+	
75	M21.9	$C_{22}H_{22}O_{10}$	447.1286	-1.5	16.73	137.0808 $C_{15}H_{13}O_4^+$	
76	22: glycyrrhetinic acid	$C_{30}H_{46}O_4$	471.3469	0.5	27.78	471.3469 $C_{30}H_{47}O_4^+$	P
77	M22.1	$C_{36}H_{54}O_{10}$	647.3790	-0.7	21.2	453.3363 $C_{30}H_{45}O_3^+$	
78	M22.2	$C_{42}H_{62}O_{16}$	823.4111	0.9	21.22	453.3363 $C_{30}H_{45}O_3^+$	
79	M22.3	$C_{38}H_{56}O_{11}$	689.3895	2.5	21.63	689.3895 $C_{38}H_{57}O_{11}^+$ 345.1544 $C_{16}H_{25}O_8^+$	
80	M22.4	$C_{35}H_{56}O_{10}$	637.3946	4	32.52	637.3946 $C_{35}H_{57}O_{10}^+$	
81	M22.5	$C_{34}H_{55}NO_6$	574.4102	3	31.48	574.4102 $C_{34}H_{56}NO_6^+$ 455.3884 $C_{31}H_{51}O_2^+$	M
82	M22.6	$C_{31}H_{48}O_4$	485.3625	0.5	30.71	485.3625 $C_{31}H_{49}O_4^+$ 331.2268 $C_{21}H_{31}O_3^+$	
83	M22.7	$C_{32}H_{53}NO_5$	532.3997	1	24.25	532.3997 $C_{32}H_{54}NO_5^+$ 156.0655 $C_7H_{10}NO_3^+$ 170.0812 $C_8H_{12}NO_3^+$ 211.2056 $C_{14}H_{27}O^+$ 532.3997 $C_{32}H_{54}NO_5^+$	
84	23: macedonic acid	$C_{30}H_{46}O_4$	471.3469	0.2	21.22	471.3469 $C_{30}H_{47}O_4^+$	P

85	M23.1	$C_{36}H_{54}O_{10}$	647.3790	-0.7	21.2	453.3363 $C_{30}H_{45}O_3^+$ 435.3258 $C_{30}H_{43}O_2^+$	
86	M23.2	$C_{36}H_{56}O_{10}$	649.3946	-0.1	21	133.0495 $C_5H_9O_4^+$ 177.0394 $C_6H_9O_6^+$	
87	M23.3	$C_{29}H_{44}O_4$	457.3312	-0.7	25.73	411.3258 $C_{28}H_{43}O_2^+$ 187.1329 $C_{10}H_{19}O_3^+$	
88	M23.4	$C_{30}H_{46}O_4$	471.3469	-1	27.77	471.3469 $C_{30}H_{47}O_4^+$ 317.2111 $C_{20}H_{29}O_3^+$	
89	M23.5	$C_{35}H_{52}O_{10}$	633.3633	-1.8	29.44	457.3312 $C_{29}H_{45}O_4^+$ 633.3633 $C_{35}H_{53}O_{10}^+$	M
90	M23.6	$C_{31}H_{48}O_4$	485.3625	0.5	30.71	485.3625 $C_{31}H_{49}O_4^+$ 331.2268 $C_{21}H_{31}O_3^+$	
91	M23.7	$C_{31}H_{48}O_4$	485.3625	0.5	30.71	485.3625 $C_{31}H_{49}O_4^+$ 331.2268 $C_{21}H_{31}O_3^+$	
92	M23.8	$C_{29}H_{46}O_4$	459.3469	-0.5	28.55	423.3258 $C_{29}H_{43}O_2^+$ 459.3469 $C_{29}H_{47}O_4^+$ 157.0859 $C_8H_{15}O_3^+$ 171.1016 $C_9H_{15}O_3^+$	
93	M23.9	$C_{36}H_{56}O_{10}$	649.3946	-0.1	21	133.0495 $C_5H_9O_4^+$ 177.0394 $C_6H_9O_6^+$	
94	24: glycycoumarin	$C_{21}H_{20}O_6$	369.1333	0.3	21.09	313.0707 $C_{17}H_{13}O_6^+$ 271.0601 $C_{15}H_{11}O_5^+$	P
95	M24.1	$C_{21}H_{26}O_7$	391.1751	2.9	29.76	149.0233 $C_8H_5O_3^+$ 123.0441 $C_7H_7O_2^+$ 139.0390 $C_7H_7O_3^+$ 391.1751 $C_{21}H_{27}O_7^+$	
96	M24.2	$C_{22}H_{28}O_7$	405.1908	-0.9	1.24	301.1071 $C_{17}H_{17}O_5^+$	
97	M24.3	$C_{22}H_{22}O_6$	383.1489	0.4	24.45	327.0863 $C_{18}H_{15}O_6^+$	M
98	M24.4	$C_{33}H_{36}O_{18}$	721.1974	1.1	15.88	313.0918 $C_{14}H_{17}O_8^+$	
99	M24.5	$C_{24}H_{26}O_6$	411.1802	2.6	9.44	411.1802 $C_{24}H_{27}O_6^+$	
100	25: glycyrrhetinic acid 3-O-mono-beta-D-glucuronide	$C_{36}H_{54}O_{10}$	647.3790	0.9	21.22	453.3363 $C_{30}H_{45}O_3^+$	P
101	M25.1	$C_{30}H_{46}O_4$	471.34690	-1	27.77	471.3469 $C_{30}H_{47}O_4^+$ 317.2111 $C_{20}H_{29}O_3^+$ 235.1693 $C_{15}H_{23}O_2^+$	
102	M25.2	$C_{36}H_{54}O_{10}$	647.3790	-0.7	21.2	453.3363 $C_{30}H_{45}O_3^+$ 435.3258 $C_{30}H_{43}O_2^+$	M
103	M25.3	$C_{42}H_{62}O_{16}$	823.4111	0.9	21.22	453.3363 $C_{30}H_{45}O_3^+$ 647.3790 $C_{36}H_{55}O_{10}^+$	
104	M25.4	$C_{36}H_{56}O_{10}$	649.3946	-0.1	21	133.0495 $C_5H_9O_4^+$ 177.0394 $C_6H_9O_6^+$	
105	M25.5	$C_{36}H_{56}O_{10}$	649.3946	-0.1	21	133.0495 $C_5H_9O_4^+$ 177.0394 $C_6H_9O_6^+$	
106	26: retrochalcone	$C_{16}H_{14}O_4$	271.0965	0	16.74	137.0597 $C_8H_9O_2^+$	P
107	M26.1	$C_{16}H_{18}O_4$	275.1278	-0.5	19.28	133.0648 $C_9H_9O^+$ 105.0335 $C_7H_5O^+$ 275.1278 $C_{16}H_{19}O_4^+$ 151.0754 $C_9H_{11}O_2^+$ 179.1067 $C_{11}H_{15}O_2^+$	
108	M26.2	$C_{16}H_{16}O_4$	273.1121	-0.9	15.5	137.0597 $C_8H_9O_2^+$ 123.0441 $C_7H_7O_2^+$ 163.0754 $C_{10}H_{11}O_2^+$	
109	M26.3	$C_{16}H_{16}O_4$	273.1121	-0.9	15.5	137.0597 $C_8H_9O_2^+$ 123.0441 $C_7H_7O_2^+$ 163.0754 $C_{10}H_{11}O_2^+$ 103.0178 $C_7H_3O^+$	
110	M26.4	$C_{30}H_{42}O_{16}$	659.2546	-1.3	28.82	479.1548 $C_{23}H_{27}O_{11}^+$	M
111	M26.5	$C_{18}H_{20}O_4$	301.1434	-4.8	28.09	301.1434 $C_{18}H_{21}O_4^+$	
112	M26.6	$C_{18}H_{20}O_4$	301.1434	-4.8	28.09	301.1434 $C_{18}H_{21}O_4^+$	
113	M26.7	$C_{23}H_{30}O_{10}$	467.1912	-4.9	7.99	125.0597 $C_7H_9O_2^+$	

114	27: licoarylcoumarin	$C_{21}H_{20}O_6$	369.1333	0.3	21.09	313.1071 $C_{18}H_{17}O_5^+$ 271.0601 $C_{15}H_{11}O_5^+$	P
115	M27.1	$C_{21}H_{26}O_{10}S$	471.1320	-2.8	23.62	171.1380 $C_{10}H_{19}O_2^+$	
116	M27.2	$C_{21}H_{26}O_7$	391.1751	2.9	29.76	149.0233 $C_8H_5O_3^+$ 123.0441 $C_7H_7O_2^+$ 139.0390 $C_7H_7O_3^+$ 391.1751 $C_{21}H_{27}O_7^+$	M
117	M27.3	$C_{22}H_{22}O_6$	383.1489	0.4	24.45	327.1227 $C_{19}H_{19}O_5^+$	
118	28: 4H-1-Benzopyran-4-one, 3-[4,6-dihydroxy-2-methoxy-3-(3-methyl-2-buten-1-yl)phenyl]-7-hydroxy-	$C_{21}H_{20}O_6$	369.1333	0.3	21.09	313.0707 $C_{17}H_{13}O_6^+$ 271.0601 $C_{15}H_{11}O_5^+$	P
119	M28.1	$C_{22}H_{22}O_6$	383.1489	0.4	24.45	327.0863 $C_{18}H_{15}O_6^+$	
120	M28.2	$C_{21}H_{28}O_6$	377.1959	0.3	25.51	197.0808 $C_{10}H_{13}O_4^+$ 235.1329 $C_{14}H_{19}O_3^+$ 123.0441 $C_7H_7O_2^+$	M
121	M28.3	$C_{21}H_{28}O_6$	377.1959	0.3	25.51	197.0808 $C_{10}H_{13}O_4^+$ 235.1329 $C_{14}H_{19}O_3^+$ 123.0441 $C_7H_7O_2^+$	
122	M28.4	$C_{24}H_{26}O_6$	411.1802	2.6	9.44	411.1802 $C_{24}H_{27}O_6^+$	
123	29: tetradecanoic acid	$C_{14}H_{28}O_2$	229.2162	-0.5	30.61	117.0910 $C_6H_{13}O_2^+$	P
124	M29.1	$C_{14}H_{26}O_4$	259.1904	0.6	27.6	101.0597 $C_5H_9O_2^+$ 111.0804 $C_7H_{11}O^+$ 129.0910 $C_7H_{13}O_2^+$	
125	M29.2	$C_{14}H_{28}O_6S$	325.1679	4.1	25.86	139.1117 $C_9H_{15}O^+$ 125.0961 $C_8H_{13}O^+$	
126	M29.3	$C_{14}H_{28}O_6S$	325.1679	4.1	25.86	139.1117 $C_9H_{15}O^+$ 125.0961 $C_8H_{13}O^+$	M
127	M29.4	$C_{14}H_{28}O_9S_2$	405.1248	-1.5	1.21	153.1274 $C_{10}H_{17}O^+$ 211.2056 $C_{14}H_{27}O^+$	
128	M29.5	$C_{14}H_{28}O_9S_2$	405.1248	-1.5	1.21	153.1274 $C_{10}H_{17}O^+$ 211.2056 $C_{14}H_{27}O^+$	

P stands for Prototype compound, M stands for metabolite.
