# Supplementary figures and images for: Screening of Biomarkers and Quality Control of Shaoyao Gancao Decoction Using UPLC-MS/MS Combined with Network Pharmacology and Molecular Docking Technology
Source: Evid Based Complement Alternat Med. 2022 Nov 29;2022:2442681. doi: 10.1155/2022/2442681 (PMC9726270; doi:10.1155/2022/2442681)

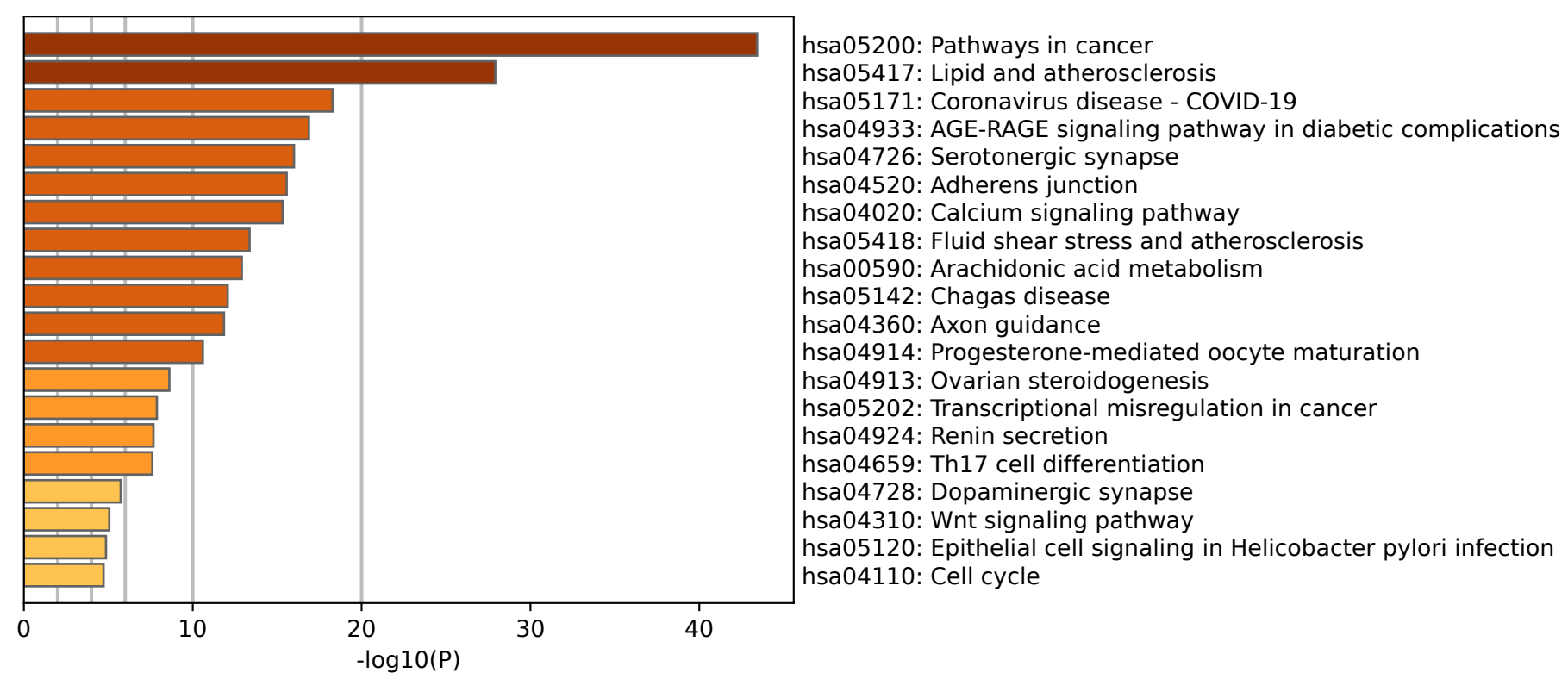

Supplement: Supplementary Materials — Table 1: Binding energies of representative compounds and targets. Table 2. 128 blood absorbed components. Figure 1: KEGG analysis of potential target genes of SGD, top 20 clusters of KEGG. Figure 2: GO analysis of potential target genes of the SGD. [file 2442681.f1.zip › Supplemental Figure S1.pdf]

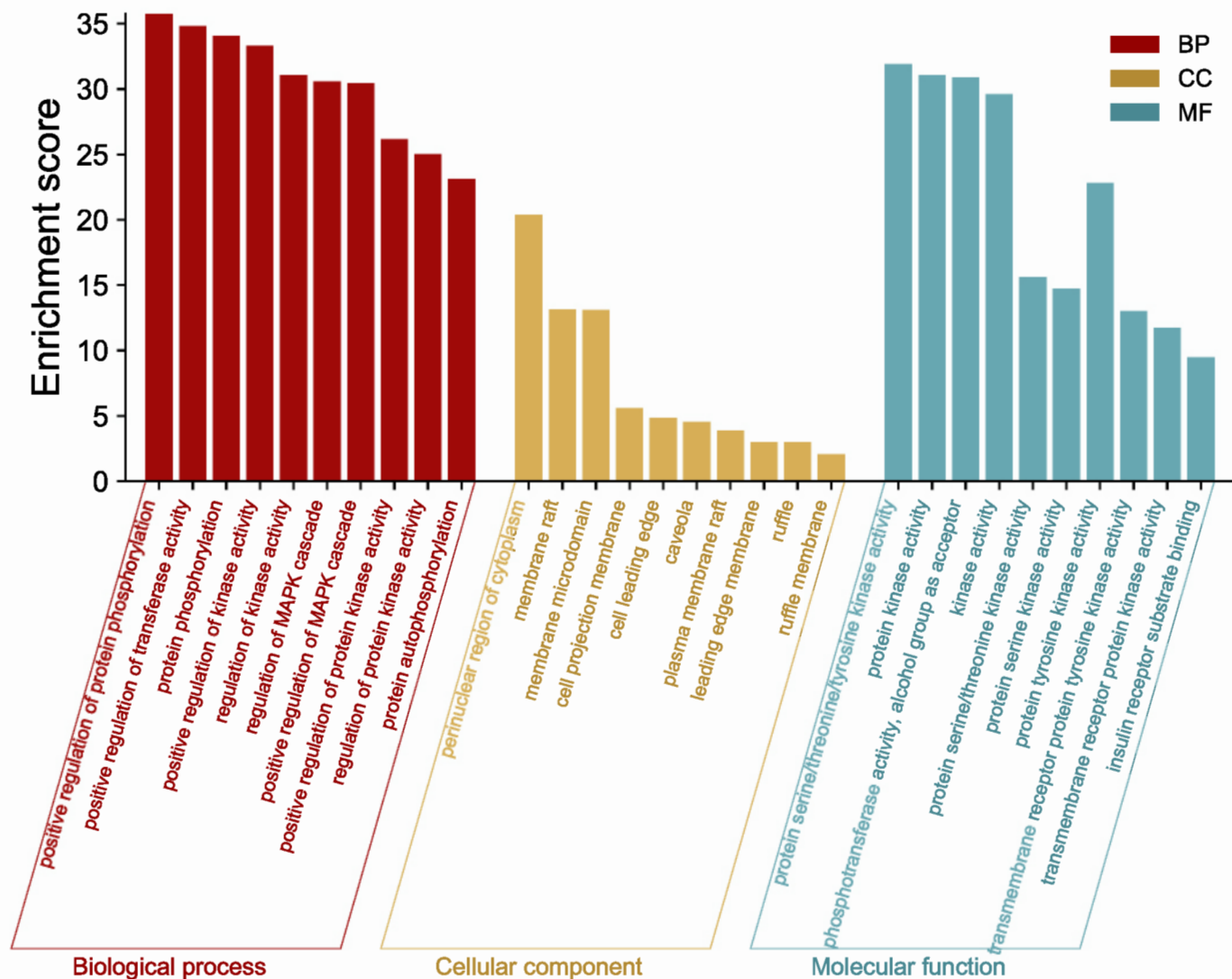

Supplement: Supplementary Materials — Table 1: Binding energies of representative compounds and targets. Table 2. 128 blood absorbed components. Figure 1: KEGG analysis of potential target genes of SGD, top 20 clusters of KEGG. Figure 2: GO analysis of potential target genes of the SGD. [file 2442681.f1.zip › Supplemental Figure S2.pdf]
